# Doubling of Asymptotically Flat Half-spaces and the Riemannian Penrose Inequality

**DOI:** 10.1007/s00220-023-04635-7

**Published:** 2023-01-31

**Authors:** Michael Eichmair, Thomas Koerber

**Affiliations:** Faculty of Mathematics, University of Vienna, Oskar-Morgenstern-Platz 1, 1090 Vienna, Austria

## Abstract

Building on previous works of Bray, of Miao, and of Almaraz, Barbosa, and de Lima, we develop a doubling procedure for asymptotically flat half-spaces (*M*, *g*) with horizon boundary $\Sigma \subset M$ and mass $m\in {\mathbb {R}}$. If $3\le \dim (M)\le 7$, (*M*, *g*) has non-negative scalar curvature, and the boundary $\partial M$ is mean-convex, we obtain the Riemannian Penrose-type inequality

$$\begin{aligned} m\ge \left( \frac{1}{2}\right) ^{\frac{n}{n-1}}\,\left( \frac{|\Sigma |}{\omega _{n-1}}\right) ^{\frac{n-2}{n-1}} \end{aligned}$$

as a corollary. Moreover, in the case where $\partial M$ is not totally geodesic, we show how to construct local perturbations of (*M*, *g*) that increase the scalar curvature. As a consequence, we show that equality holds in the above inequality if and only if the exterior region of (*M*, *g*) is isometric to a Schwarzschild half-space. Previously, these results were only known in the case where $\dim (M)=3$ and $\Sigma$ is a connected free boundary hypersurface.

## Introduction

Let (*M*, *g*) be a connected, complete Riemannian manifold of dimension $3\le n\le 7$ with integrable scalar curvature *R*(*g*) and non-compact boundary $\partial M$ with integrable mean curvature $H(\partial M,g)$. Here, $H(\partial M,g)$ is computed as the divergence along $\partial M$ of the normal $-\nu (\partial M,g)$ pointing out of *M*.

We say that (*M*, *g*) is an asymptotically flat half-space if there is a number $\tau >(n-2)/2$ and a non-empty compact subset of *M* whose complement is diffeomorphic to $\{x\in {\mathbb {R}}^n_+:|x|_{{\bar{g}}}>1\}$ such that, in this so-called asymptotically flat chart, as $x\rightarrow \infty$,

1 $$\begin{aligned} |g-{\bar{g}}|_{{\bar{g}}}+|x|_{{\bar{g}}}\,|D({\bar{g}})g|_{{\bar{g}}}+|x|^2_{\bar{g}}\,|D^2({\bar{g}})g|_{{\bar{g}}}=O(|x|_{{\bar{g}}}^{-\tau }). \end{aligned}$$

Here, ${\mathbb {R}}^n_+=\{x\in {\mathbb {R}}^n:x^n\ge 0\}$ is the upper half-space and ${\bar{g}}$ the Euclidean metric.

Escobar has studied asymptotically flat half-spaces in the context of the Yamabe problem for compact Riemannian manifolds with boundary; see [17] and also the related works of Brendle [9] and of Brendle and Chen [10]. Almaraz [1, pp. 2628–2629] and Almaraz, Barbosa, and de Lima [2, p. 674] have studied asymptotically flat half-space in detail and associated to them a global geometric invariant called the mass. This mass, whose definition is attributed to Marques on [2, p. 677], is given by

2 $$\begin{aligned} m(g)= & {} \frac{1}{2\,(n-1)\,\omega _{n-1}}\,\lim _{\lambda \rightarrow \infty }\lambda ^{-1}\,\bigg (\sum _{i,\,j=1}^n\int _{ {{\mathbb {R}}}^n_+\cap {S}^{n-1}_\lambda (0)}x^i\, \big [(\partial _jg)(e_i,e_j)-(\partial _ig)(e_j,e_j)\big ]\,\textrm{d}\mu ({\bar{g}})\nonumber \\ {}{} & {} \qquad +\sum _{i=1}^{n-1}\int _{({\mathbb {R}}^{n-1}\times \{0\})\cap S^{n-1}_\lambda (0)} x^i\,g(e_i,e_n)\,\textrm{d}l({\bar{g}}) \bigg ) \end{aligned}$$

where the integrals are computed in the asymptotically flat chart. Here, $\omega _{n-1}=|\{x\in {\mathbb {R}}^n:|x|_{{\bar{g}}}=1\}|_{{\bar{g}}}$ denotes the Euclidean area of the $(n-1)$-dimensional unit sphere and $e_1,\dots ,e_n$ is the standard basis of ${\mathbb {R}}^n$. In analogy with the work [34] of Schoen on closed manifolds, Escobar has established a connection between the magnitude of the Yamabe-invariant of a compact manifold with boundary and the sign of the mass (2) of an associated asymptotically flat half-space in [17]. Almaraz, Barbosa, and de Lima have showed that the mass (2) is a geometric invariant and, in fact, non-negative provided that (*M*, *g*) satisfies suitable energy conditions. As noted in [2, p. 675], previous results in this direction had been obtained by Escobar [17, Appendix] and by Raulot [33, Theorem 23].

### Theorem 1

([2, Theorem 1.3]). Let (*M*, *g*) be an asymptotically flat half-space of dimension $3\le n\le 7$ such that $R(g)\ge 0$ and $H(\partial M,g)\ge 0$. Then $m(g)\ge 0$. Moreover, $m(g)=0$ if and only if (*M*, *g*) is isometric to $({\mathbb {R}}^n_+,\,{\bar{g}})$.

### Remark 2

As explained in [31, §2], the assumption that $H(\partial M,g)\ge 0$ can and should be viewed as a non-negativity condition for the scalar curvature *R*(*g*) across $\partial M$ in a distributional sense; see also [7, p. 207]. We note that this condition also has a natural physical interpretation; [3, Remark 2.7].

Theorem 1 is fashioned after the positive mass theorem for asymptotically flat initial data for the Einstein field equations, which has been proved by Schoen and Yau [36] using minimal surface techniques and by Witten [40] using certain solutions of the Dirac equation. In the presence of a so-called outermost minimal surface in the initial data set, a heuristic argument due to Penrose [32] suggests a stronger, quantitative version of the positive mass theorem which has been termed the Riemannian Penrose inequality. This inequality has been verified by Huisken and Ilmanen in [21] in dimension $n=3$ when the outermost minimal surface is connected, by Bray in [7] in the case of a possibly disconnected outermost minimal surfaces, and by Bray and Lee in [8] in the case where $3\le n\le 7$ and the outermost minimal surface may be disconnected. We provide more details on asymptotically flat manifolds without boundary, the positive mass theorem, and the Riemannian Penrose inequality in Appendix A.

Almaraz, de Lima, and Mari [3] have studied the mass (2) of initial data sets with non-compact boundary in a spacetime setting; see also the recent survey [15] of de Lima. Moreover, they argue that, in the presence of an outermost minimal surface, a Riemannian Penrose-type inequality should hold for asymptotically flat half-spaces as well; see [3, Remark 5.6].

**Fig. 1:**
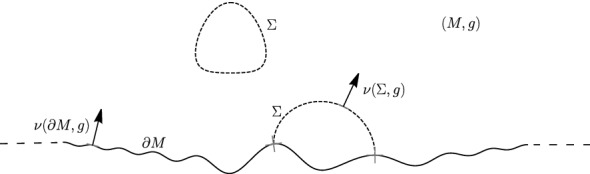
An illustration of an asymptotically flat half-space (*M*, *g*) with horizon boundary. $\partial M$ is illustrated by the solid black line. The horizon boundary $\Sigma$, consisting of a free boundary hypersurface and a closed hypersurface, is illustrated by the two dotted lines. The unit normals $\nu (M,g)$ and $\nu (\Sigma ,g)$ are showed by the two arrows

To describe recent results in this direction, we recall the following definitions from [23]; see Fig. 1. Let $\Sigma \subset M$ be a compact separating hypersurface satisfying $\Sigma \cap \partial M=\partial \Sigma$ with normal $\nu (\Sigma ,g)$ pointing towards the closure $M(\Sigma )$ of the non-compact component of $MP{\setminus } \Sigma$. We call a component $\Sigma ^0$ of $\Sigma$ a free boundary hypersurface if $\partial \Sigma ^0\ne \emptyset$ and $\nu (\Sigma )(x)\in T_x\partial M$ for every $x\in \partial \Sigma ^0$. If $\partial \Sigma ^0=\emptyset$, we call $\Sigma ^0$ a closed hypersurface.

We say that an asymptotically flat half-space (*M*, *g*) has horizon boundary $\Sigma \subset M$ if $\Sigma$ is a non-empty compact minimal hypersurface with the following properties.

- The connected components of $\Sigma$ are either free boundary hypersurfaces or closed hypersurfaces.
- Every minimal free boundary hypersurfaces or minimal closed hypersurfaces in $M(\Sigma )$ is a component of $\Sigma$.

### Remark 3

The proof of [23, Lemma 2.3] shows that every asymptotically flat half-space (*M*, *g*) of dimension $3\le n\le 7$ with $H(\partial M,g)\ge 0$ either has a unique horizon boundary $\Sigma \subset M$ or contains no compact minimal hypersurfaces.

The region $M(\Sigma )$ outside of the horizon boundary is called an exterior region. The horizon $\Sigma$ is also called an outermost minimal surface. An example of an exterior region with horizon boundary is the Schwarzschild half-space of mass $m>0$ and dimension $n\ge 3$, given by

3 $$\begin{aligned} (M(\Sigma ),g)=\bigg (\left\{ x\in {\mathbb {R}}^n_+:|x|_{{\bar{g}}}\ge m^{\frac{1}{n-2}}\right\} ,\left( 1+m\,|x|_{\bar{g}}^{2-n}\right) ^\frac{4}{n-2}\,{\bar{g}}\bigg ) \end{aligned}$$

where $\Sigma =\{x\in {\mathbb {R}}^n_+:|x|_{\bar{g}}=m^{\frac{1}{n-2}}\}$; see [2, Remark 3.10] and [23, §2].

The second-named author has recently proved the following Riemannian Penrose-type inequality for asymptotically flat half-spaces whose horizon boundary is a connected free boundary hypersurface.

### Theorem 4

([23, Theorem 1.2]). Let (*M*, *g*) be an asymptotically flat half-space of dimension $n=3$ with horizon boundary $\Sigma \subset M$ such that the following three conditions hold.

- $$R(g)\ge 0$$ in $$M(\Sigma )$$.
- $$H(\partial M,g)\ge 0$$ on $$ M(\Sigma )\cap \partial M$$.
- $\Sigma$ is a connected free boundary hypersurface.

Then

$$\begin{aligned} m(g)\ge \sqrt{\frac{|\Sigma |_g}{32\,\pi }} \end{aligned}$$

with equality if and only if $(M(\Sigma ),g)$ is isometric to a Schwarzschild half-space (3).

### Remark 5

Previous results in direction of Theorem 4 for asymptotically flat half-spaces arising as certain graphical hypersurfaces in Euclidean space had been obtained by Barbosa and Meira [5].

### Remark 6

The method of weak free boundary inverse mean curvature flow employed in the proof of Theorem 4 in [23] had been studied previously by Marquardt in [27]. It appears to the authors of this paper that the scope of this method is essentially limited to the case where $n=3$ and $\Sigma$ is a connected free boundary hypersurface; see [23, p. 16].

### Remark 7

Theorem 4 is related to a Penrose-type inequality for so-called asymptotically flat support surfaces conjectured by Huisken and studied by Volkmann; see [39, p. 38] and [23, Lemma 2.1].

### Outline of our results

Comparing Theorem 4 with the Riemannian Penrose inequality for asymptotically flat manifolds, stated here as Theorem 41, suggests that the assumptions that $n=3$ and that $\Sigma$ be a connected free boundary hypersurface in Theorem 4 are not necessary. The goal of this paper is to address this conjecture using a strategy different from that in [23]. In fact, we demonstrate how the gluing method developed by Miao in [31], which in turn expands on an idea of Bray [7], can be used to develop a doubling procedure for asymptotically flat half-spaces that reduces the Riemannian Penrose inequality for asymptotically flat half-spaces to the Riemannian Penrose inequality for asymptotically flat manifolds.

For the statement of Theorem 8, recall from Appendix A the definition of an asymptotically flat manifold $({\tilde{M}},{\tilde{g}})$, of its mass ${\tilde{m}}$, of its horizon boundary ${\tilde{\Sigma }}$, and of the exterior region $\tilde{M}({\tilde{\Sigma }})$.

#### Theorem 8

Let (*M*, *g*) be an asymptotically flat half-space of dimension $3\le n\le 7$ with horizon boundary $\Sigma \subset M$ such that the following two conditions hold.

- $$R(g)\ge 0$$ in $$M(\Sigma )$$.
- $$H(\partial M,g)\ge 0$$ on $$ M(\Sigma )\cap \partial M$$.

Let $\varepsilon >0$. There exists an asymptotically flat manifold $({\tilde{M}},{\tilde{g}})$ with horizon boundary $\tilde{\Sigma }\subset {\tilde{M}}$ such that

- $$R({\tilde{g}})\ge 0$$ in $${\tilde{M}}({\tilde{\Sigma }})$$,
- $$|{\tilde{m}}({\tilde{g}})- 2\,m(g)|< \varepsilon $$, and
- $$||{\tilde{\Sigma }}|_{{\tilde{g}}}-2\,|\Sigma |_g|< \varepsilon $$.

#### Remark 9

Gluing constructions related to the one used in the proof of Theorem 8 have also been studied by Miao and McCormick in [28] and by Lu and Miao in [26].

Combining Theorem 8 with Theorem 41, we are able to extend the Riemannian Penrose inequality for asymptotically flat half-spaces to dimensions less than 8 and to horizon boundaries that may be disconnected.

#### Corollary 10

Let (*M*, *g*) be an asymptotically flat half-space of dimension $3\le n\le 7$ with horizon boundary $\Sigma \subset M$ such that the following two conditions hold.

- $$R(g)\ge 0$$ in $$M(\Sigma )$$.
- $$H(\partial M,g)\ge 0$$ on $$ M(\Sigma )\cap \partial M$$.

Then

4 $$\begin{aligned} m(g)\ge \left( \frac{1}{2}\right) ^{\frac{n}{n-1}}\,\left( \frac{|\Sigma |_g}{\omega _{n-1}}\right) ^{\frac{n-2}{n-1}}. \end{aligned}$$

#### Remark 11

Carlotto and Schoen have showed in [12, Theorem 2.3] that there is an abundance of asymptotically flat Riemannian manifolds with non-negative scalar curvature that contain a Euclidean half-space isometrically. Note that Corollary 10 shows that the Riemannian Penrose inequality, stated here as Theorem 41, can be localized to the geometrically non-trivial part of such initial data.

The approximation argument used to prove Corollary 10 cannot be applied to characterize the case of equality in (4) directly. Yet, we observe that (*M*, *g*) can be locally perturbed to increase the scalar curvature near non-umbilical points of the boundary $\partial M$. Combining this insight with a variational argument used by Schoen and Yau [36] to characterize the case of equality in the positive mass theorem, we are able to prove the following rigidity result.

#### Theorem 12

Let (*M*, *g*) be an asymptotically flat half-space of dimension $3\le n\le 7$ with horizon boundary $\Sigma \subset M$ such that the following two conditions hold.

- $$R(g)\ge 0$$ in $$M(\Sigma )$$.
- $$H(\partial M,g)\ge 0$$ on $$ M(\Sigma )\cap \partial M$$.

Assume that

$$\begin{aligned} m(g)=\left( \frac{1}{2}\right) ^{\frac{n}{n-1}}\,\left( \frac{|\Sigma |_g}{\omega _{n-1}}\right) ^{\frac{n-2}{n-1}}. \end{aligned}$$

Then $(M(\Sigma ),g)$ is isometric to a Schwarzschild half-space (3).

#### Remark 13

Bray and Lee [8] have proved rigidity of the Riemannian Penrose inequality for asymptotically flat manifolds $({\tilde{M}},{\tilde{g}})$ of dimension $3\le n\le 7$ under the additional assumption that $({\tilde{M}},{\tilde{g}})$ be spin; see Theorem 41. Building on previous work [29] by McFeron and Székelyhidi, Lu and Miao [26, Theorem 1.1] have showed that the spin assumption can be dispensed with. Using the techniques developed in this paper, we are able to give a short alternative proof of this fact; see Theorem 39.

#### Remark 14

We survey several important contributions to scalar curvature rigidity results preceding Theorem 12 in Appendix F.

#### Remark 15

For the proofs of Theorem 8, Corollary 10, and Theorem 12, it is sufficient to require the metric *g* to be of class $C^{2,\alpha }$. For the sake of readability, we will assume throughout that *g* is smooth.

### Outline of the proof

Let (*M*, *g*) be an asymptotically flat half-space with horizon boundary $\Sigma \subset M$ and suppose that $R(g)\ge 0$ in $M(\Sigma )$ and $H(\partial M,g) \ge 0$ on $M(\Sigma )\cap \partial M$. The basic idea to prove Theorem 8 is to consider the double $({\tilde{M}}, {\tilde{g}})$ of (*M*, *g*) obtained by reflection across $\partial M$. The metric ${\tilde{g}}$ is only $C^0$ across $\partial M$. The condition $H(\partial M,g)\ge 0$ suggests that the scalar curvature of ${\tilde{g}}$ is non-negative in a distributional sense; see Remark 2. Moreover, since $\Sigma \subset M$ is an outermost minimal surface that intersects $\partial M$ orthogonally, its double ${\tilde{\Sigma }}\subset \tilde{M}$ is an outermost minimal surface without boundary.

The difficulty in rendering this heuristic argument rigorous is that $({\tilde{M}},{\tilde{g}})$ needs to be smoothed in a way that allows us to keep track of the mass, the horizon boundary, and the relevant energy conditions all at the same time. To this end, we first adapt an approximation procedure developed by Almaraz, Barbosa, and de Lima in [2, Proposition 4.1] to arrange that *g* is scalar flat and conformally flat at infinity and that $\partial M$ is totally geodesic at infinity; see Proposition 16. In particular, the reflected metric ${\tilde{g}}$ is $C^2$ outside of a bounded open set $W\subset M$. Moreover, using a local conformal perturbation of the metric, we may arrange that $\Sigma$ is strictly mean convex; see Lemma 20.

Next, we smooth ${\tilde{g}}$ near $W\cap \partial M$ using a technique developed by Miao in [31]. In this step, the mean convexity of $\partial M$ ensures that the scalar curvature of the smoothed metric remains uniformly bounded from below near $\partial M$; see Lemma 24. Moreover, we show that the strict mean convexity of ${\tilde{\Sigma }}$ is not affected by this procedure; see Lemma 25.

By a conformal transformation similar to that developed by Miao in [31, §4], building in turn on [36, Lemma 3.3], we remove the small amount of negative scalar curvature that may have been created close to $\partial M$ in the approximation process. This conformal transformation only changes the mass of the smoothed manifold by a small amount; see Proposition 31. Finally, using ${\tilde{\Sigma }}$ as a barrier, it follows that the smoothed metric has horizon boundary. Since $\Sigma \subset M$ is area-minimizing, it follows that the area of the new horizon boundary is at least as large as that of ${\tilde{\Sigma }}$; see Lemma 32. This is how we obtain Theorem 8.

To prove Theorem 12, we first construct a global conformal perturbation of $(M(\Sigma ),g)$ that preserves the conditions $R(g)\ge 0$ and $H(\partial M,g)\ge 0$, strictly decreases *m*(*g*) unless $R(g)=0$, and which changes the area of $\Sigma$ only marginally. Second, if the second fundamental form $h(\partial M,g)$ of $\partial M$ does not vanish, we construct a local perturbation of $(M(\Sigma ),g)$ that increases *R*(*g*), preserves the condition $H(\partial M,g)\ge 0$, and changes neither *m*(*g*) nor $|\Sigma |_g$. We note that a perturbation with these properties could not possibly be conformal; it has to be fine-tuned to the geometry of $\partial M$. Consequently, if equality in (4) holds, then $\partial M$ is totally geodesic and the double $({\tilde{M}},{\tilde{g}})$ is $C^2$-asymptotically flat. Theorem 12 now follows from Theorem 41.

## Reduction to Conformally Flat Ends

In this section, we assume that (*M*, *g*) is an asymptotically flat half-space of dimension $3 \le n \le 7$ and decay rate $\tau >(n-2)/2$. We also assume that (*M*, *g*) has horizon boundary $\Sigma \subset M$ and that $R(g)\ge 0$ in $M(\Sigma )$ and $H(\partial M, g)\ge 0$ on $M(\Sigma )\cap \partial M$.

The goal of this section is to approximate the Riemannian metric *g* by a sequence $\{g_i\}_{i=1}^\infty$ of Riemannian metrics $g_i$ on *M* that are scalar flat, conformally flat, and such that $h(\partial M, g_i)=0$ outside of some compact set.

Here and below, $\Sigma$ and $\partial M$ are oriented by their unit normal vectors $\nu (\Sigma ,g)$ and $\nu (\partial M,g)$ pointing towards $M(\Sigma )$. $H(\Sigma ,g)$ and $H(\partial M,g)$ are computed as the divergence of $-\nu (\Sigma ,g)$ along $\Sigma$ and the divergence of $-\nu (\partial M,g)$ along $\partial M$, respectively.

### Proposition 16

Let $\tau '\in {\mathbb {R}}$ be such that $(n-2)/2<\tau '<\tau$. There exist sequences $\{g_i\}_{i=1}^\infty$ of Riemannian metrics $g_i$ on *M* and $\{K_i\}_{i=1}^\infty$ of compact sets $K_i\subset M$ such that $(M, g_i)$ is an asymptotically flat half-space with horizon boundary $\Sigma _i\subset M(\Sigma )$ and such that the following properties hold.

- $(M,g_i)$ is conformally flat in $M{\setminus } K_i$.
- $$R(g_i)=0$$ in $$M{\setminus } K_i$$.
- $$h(\partial M,g_i)=0$$ on $$\partial M{\setminus } K_i$$.
- $$R(g_i)\ge 0$$ in $$M(\Sigma )$$.
- $$H(\partial M,g_i)\ge 0$$ on $$ M(\Sigma )\cap \partial M$$.
- $$m(g_i)=m(g)+o(1)$$ as $$i\rightarrow \infty $$.
- $$|\Sigma _i|_{g_i}=|\Sigma |_g+o(1)$$ as $$i\rightarrow \infty $$.
- $$g_i\rightarrow g$$ in $$C^0(M)$$ as $$i\rightarrow \infty $$.

Moreover,

5 $$\begin{aligned} \sup _{i\ge 1}\,\limsup _{x\rightarrow \infty } \left[ |x|_{\bar{g}}^{\tau }\,|g_i-{\bar{g}}|_{{\bar{g}}}+|x|^{\tau +1}_{{\bar{g}}}\,|D(\bar{g})g_i|_{{\bar{g}}}+|x|^{\tau +2}_{{\bar{g}}}\,|D^2({\bar{g}})g_i|_{\bar{g}}\right] <\infty . \end{aligned}$$

### Proof

Arguing as in the proof of [2, Proposition 4.1] but using Proposition 46 instead of [2, Proposition 3.3], we obtain a sequence $\{g_i\}_{i=1}^\infty$ of Riemannian metrics $g_i$ on *M* and a sequence $\{K_i\}_{i=1}^\infty$ of compact sets $K_i\subset M$ exhausting *M* such that the following properties hold.

- $g_i$ is conformally flat in $M{\setminus } K_i$.
- $$R(g_i)=0$$ in $$ M{\setminus } K_i$$.
- $$h(\partial M,g_i)=0$$ on $$\partial M{\setminus } K_i$$.
- $$R(g_i)\ge 0$$ in $$M(\Sigma )$$.
- $$H(\partial M,g_i)\ge 0$$ on $$ M(\Sigma )\cap \partial M$$.
- $$H(\Sigma ,g_i)=0$$ on $$\Sigma $$.
- $$m(g_i)=m(g)+o(1)$$ as $$i\rightarrow \infty $$.

Moreover, $g_i\rightarrow g$ in $C^0(M)$, $g_i\rightarrow g$ in $C_{loc}^2(M)$, and (5) holds.

By (5), there is $\lambda _0>1$ such that the hemispheres ${\mathbb {R}}^n_+\cap S_\lambda (-1/2\,\lambda \,e_n)$ have negative mean curvature with respect to $g_i$ and meet $\partial M$ at an acute angle with respect to $g_i$ provided that $\lambda \ge \lambda _0$ and *i* is sufficiently large. We consider the class of all embedded hypersurfaces of $M(\Sigma )$ that are homologous to $\Sigma$ in $M(\Sigma )$ and whose boundary is contained in $\partial M$ and homotopy equivalent to $\partial \Sigma$ in $M(\Sigma ) \cap \partial M$. Since $H(\Sigma ,g_i)=0$, it follows from [30, Theorem 1] that there is an outermost minimal hypersurface $\Sigma _i\subset M(\Sigma )$ that is homologous to $\Sigma$ in $M(\Sigma )$, whose boundary is homotopy equivalent to $\partial \Sigma$ in $M(\Sigma )\cap \partial M$, and whose components are either free boundary hypersurfaces or closed hypersurfaces. Moreover,

6 $$\begin{aligned} \sup _{i\ge 1} |\Sigma _i|_{g_i}<\infty . \end{aligned}$$

Recall from [23, Lemma 2.3] that $\Sigma$ is area-minimizing with respect to *g* in $M(\Sigma )$. Consequently, as $i\rightarrow \infty$,

$$\begin{aligned} |\Sigma |_{g}\le |\Sigma _i|_{g}\le (1+o(1))| \Sigma _i|_{g_i}. \end{aligned}$$

Finally, using (6), the curvature estimate [23, Lemma 3.3], and standard elliptic theory, it follows that, passing to another subsequence if necessary, $\{\Sigma _i\}_{i=1}^\infty$ converges to a minimal surface $\Sigma _0\subset M(\Sigma )$ with respect to *g* in $C^{1,\alpha }(M)$ and smoothly away from $\partial M$, possibly with finite multiplicity. Since $M(\Sigma )$ is an exterior region, it follows that $\Sigma _0=\Sigma$. Since $\Sigma _i$ is area-minimizing in $M(\Sigma _i)$ with respect to $g_i$, we obtain that, as $i\rightarrow \infty$,

$$\begin{aligned} |\Sigma _i|_{g_i}\le (1+o(1)) |\Sigma |_{g_i}\le (1+o(1))\,|\Sigma |_{g}. \end{aligned}$$

The assertion follows.

## Gluing of Asymptotically Flat Half-spaces

In this section, we assume that (*M*, *g*) is an asymptotically flat half-space of dimension $3\le n\le 7$ with horizon boundary $\Sigma \subset M$ such that the following properties are satisfied.

- (*M*, *g*) is conformally flat outside of a compact set.
- $R(g)=0$ outside of a compact set.
- $h(\partial M, g)=0$ outside of a compact set.
- $$H(\partial M,g)\ge 0$$ on $$ M(\Sigma )\cap \partial M$$.

The goal of this section is to double (*M*, *g*) by reflection across $\partial M$ and to appropriately smooth the metric of the double.

**Fig. 2:**
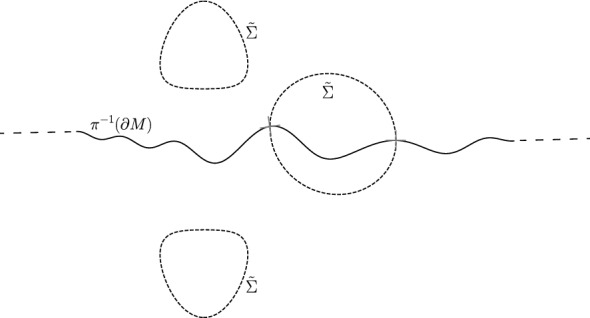
An illustration of the double $({\tilde{M}},{\tilde{g}})$ of (*M*, *g*). $({\tilde{M}},{\tilde{g}})$ is obtained by reflection across $\partial M$ which is illustrated by the solid black line. Here, $\Sigma$ has two components while ${\tilde{\Sigma }}$ has three components illustrated by the dashed lines

### Lemma 17

There is $\delta _0>0$ with the following property. The map

$$\begin{aligned} \Phi :\partial M\times [0,\delta _0)\rightarrow \{x\in M:{\text {dist}}(x,\partial M,g)<\delta _0\} \end{aligned}$$

given by

$$\begin{aligned} \Phi (y,t)=\exp (g)_y(t\,\nu (\partial M,g)(y)) \end{aligned}$$

is a diffeomorphism.

### Proof

Clearly, $\Phi$ is a local diffeomorphism and surjective. Moreover, by compactness and Lemma 45, using the fact that *g* is asymptotically flat (1), it follows that $\Phi$ is injective. $\square$

### Remark 18

It follows from Lemma 17 that there is a smooth family $\{\gamma (g)_t:t\in [0,\delta _0)\}$ of Riemannian metrics $\gamma (g)_t$ on $\partial M$ such that

$$\begin{aligned} \Phi ^*g=\gamma (g)_t+dt^2. \end{aligned}$$

Let

7 $$\begin{aligned} {\tilde{M}}=(M\times \{-1,1\})/\sim \end{aligned}$$

where

- $(x_1,\pm 1)\sim (x_2,\pm 1)$ if and only if $x_1=x_2$ and
- $(x_1,\pm 1)\sim (x_2,\mp 1)$ if and only if $x_1,\,x_2\in \partial M$ and $x_1=x_2.$

Let

8 $$\begin{aligned} \pi :{\tilde{M}}\rightarrow M\qquad \text {be given by}\qquad \pi ([(x,\pm 1)])=x \end{aligned}$$

and

$$\begin{aligned} {\tilde{\Sigma }}=\pi ^{-1}(\Sigma ); \end{aligned}$$

see Fig. 2.

We consider the map

$$\begin{aligned} {\tilde{\Phi }}:\partial M\times (-\delta _0,\delta _0)\rightarrow \{{\tilde{x}}\in {\tilde{M}}:{\text {dist}}(\pi ({\tilde{x}}),\partial M,g)<\delta _0\} \end{aligned}$$

given by

$$\begin{aligned} \qquad \tilde{\Phi }(y,t)=[(\Phi (y,|t|),{\text {sign}}(t))]. \end{aligned}$$

We obtain a smooth structure on ${\tilde{M}}$ by requiring that the map $\tilde{\Phi }$ be smooth. Moreover, given $t\in (-\delta _0,0]$, we define $\gamma (g)_{t}=\gamma (g)_{-t}$. Note that $\gamma (g)_t+dt^2$ is continuous on $\partial M\times (-\delta _0,\delta _0)$. It follows that the Riemannian metric ${\tilde{g}}$ on ${\tilde{M}}$ defined by

$$\begin{aligned} {\tilde{g}}=\pi ^*g \end{aligned}$$

is continuous across $\partial M$.

For the following lemma, recall from Appendix A the definitions (29) of an asymptotically flat metric and (30) of the mass of an asymptotically flat manifold without boundary.

### Lemma 19

${\tilde{g}}$ is of class $C^0$ and $C^2$-asymptotically flat. Moreover, the following properties hold.

- $$ {\tilde{m}}({\tilde{g}})=2\,m(g). $$
- $$ |{\tilde{\Sigma }}|_{{\tilde{g}}}=2\,|\Sigma |_{g}. $$
- ${\tilde{\Sigma }}\subset M$ is of class $C^{1,1}$.
- ${\tilde{\Sigma }}$ is area-minimizing in its homology class in ${\tilde{M}}({\tilde{\Sigma }})$ with respect to ${\tilde{g}}$.

### Proof

**Fig. 3:**
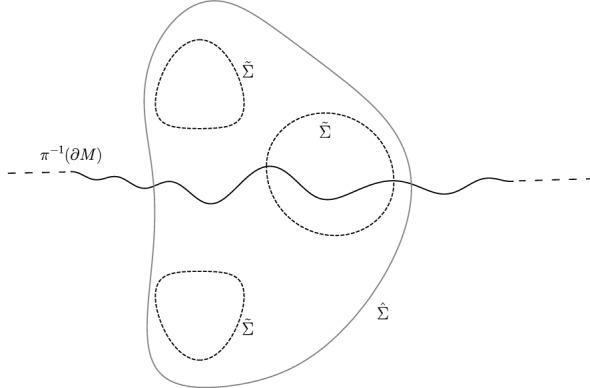
An illustration of the proof of Lemma 19. $\pi ^{-1}(\partial M)$ is depicted by the solid black line, ${\tilde{\Sigma }}$ is presented by the dashed line. Another hypersurface $\hat{\Sigma }\subset {\tilde{M}}({\tilde{\Sigma }})$ homologous to ${\tilde{\Sigma }}$ is presented by the solid gray line. Each component of $\pi (\hat{\Sigma }{\setminus } \partial M)$ is homologous to $\Sigma$ and in the same boundary homotopy class as $\Sigma$

The assertions follow from the above construction, using that (*M*, *g*) is conformally flat at infinity, that $\partial M$ is totally geodesic at infinity, that $\Sigma$ intersects $\partial M$ orthogonally, and that $\Sigma$ is area-minimizing in its homology class and boundary homotopy class in $M(\Sigma )$; see Fig. 3. $\square$

### Lemma 20

There exist a sequence $\{g^i\}_{i=1}^\infty$ of Riemannian metrics $g^i$ on *M* and a neighborhood $W\Subset M$ of $\Sigma$ such that $g^i$ and *g* are conformally equivalent, $g^i=g$ outside of *W*,  and $g^i\rightarrow g$ in $C^2(M)$. Moreover,

- $$H(\Sigma ,g^i)>0$$ and
- $$H(\partial M, g^i)\ge 0$$.

### Proof

Let $\{\psi _i\}^\infty _{i=1}$ be the sequence from Lemma 48 and let $g^i=(1+\psi _i)^{\frac{4}{n-2}}\,g$. The assertions follow from Lemmas 48 and 44. $\square$

### Lemma 21

Notation as in Lemma 20. For all $\delta _0>0$ sufficiently small, the maps

$$\begin{aligned} \Phi _i:\partial M\times [0,\delta _0)\rightarrow \{x\in M:{\text {dist}}(x,\partial M,g^i)<\delta _0\} \end{aligned}$$

given by

$$\begin{aligned} \Phi _i(y,t)=\exp (g^i)_y(t\,\nu (\partial M,g^i)(y)) \end{aligned}$$

are diffeomorphisms for all *i*. Moreover, there are smooth families $\{\gamma (g^i)_t:t\in [0,\delta _0)\}$ of Riemannian metrics $\gamma (g^i)_t$ on $\partial M$ with

$$\begin{aligned} \Phi _i^*g^i=\gamma (g^i)_t+dt^2. \end{aligned}$$

### Proof

This follows as in Lemma 17, using also that $g^i=g$ outside of *W* and that $g^i\rightarrow g$ in $C^2(M)$; see Lemma 20. $\square$

As before, we consider the maps

$$\begin{aligned} {\tilde{\Phi }}_{i}:\partial M\times (-\delta _0,\delta _0)\rightarrow \{{\tilde{x}}\in {\tilde{M}}:{\text {dist}}(\pi ({\tilde{x}}),\partial M,g^i)<\delta _0\} \end{aligned}$$

given by

$$\begin{aligned} \qquad {\tilde{\Phi }}_{i}(y,t)=[(\Phi _i(y,|t|),{\text {sign}}(t))]. \end{aligned}$$

Since *g* and $g^i$ are conformally equivalent, the maps $\tilde{\Phi }_i$ are of class $C^2$. As before, given $t\in (-\delta _0,0]$, we define $\gamma (g^i)_{t}=\gamma (g^i)_{-t}$ and obtain a continuous metric ${\tilde{g}}^i$ given by

$$\begin{aligned} {\tilde{g}}^i=\pi ^*g^i. \end{aligned}$$

To smooth the metrics ${\tilde{g}}^i$, we recall some steps from the construction in [31, §3]. To this end, let $\varphi \in C^\infty ({\mathbb {R}})$ with

$$\begin{aligned} \begin{aligned}&\bullet \qquad {\text{ spt }}(\varphi )\subset (0,1),\qquad \qquad \qquad \qquad \qquad \qquad \qquad \qquad \qquad \qquad \qquad \qquad \qquad \qquad \quad \\ {}&\bullet \qquad 0\le \varphi \le 1, \text{ and }\qquad \qquad \qquad \qquad \qquad \\ {}&\bullet \qquad \int _{0}^1\varphi (t)\,\textrm{d}t=1. \end{aligned} \end{aligned}$$

Moreover, let $\eta \in C^\infty ({\mathbb {R}})$ with

- $${\text {spt}}(\eta )\subset (-1/2,1/2)$$,
- $$\eta (t)=1/100$$ if $$|t|<1/4$$, and
- $$0\le \eta (t)\le 1/100$$ if $$1/4<|t|<1/2.$$

Let $\delta \in (0,\delta _0)$. We define $\eta _{\delta }\in C^\infty ({\mathbb {R}})$ by

$$\begin{aligned} \eta _{\delta }(t)=\delta ^{2}\,\eta (\delta ^{-1}\,t). \end{aligned}$$

Given an integer $i\ge 1$ and $t\in (-\delta _0,\delta _0)$, we define the Riemannian metric

9 $$\begin{aligned} \gamma ({\tilde{g}}^i)^\delta _t=\int _{0}^1\gamma ({\tilde{g}}^i)_{t-t\,\eta _\delta (s)}\,\varphi (s)\,\textrm{d}s \end{aligned}$$

on $$\partial M$$.

### Lemma 22

([31, Lemma 3.2]). The metric $\gamma ({\tilde{g}}^i)^\delta _t+dt^2$ is $C^2$ in $\partial M\times (-\delta _0,\delta _0)$ and agrees with $\gamma ({\tilde{g}}^i)_t+dt^2$ outside of $\partial M\times (-\delta /2,\delta /2)$.

We obtain a Riemannian metric ${\tilde{g}}^i_\delta$ on ${\tilde{M}}$ of class $C^2$ given by

10 $$\begin{aligned} {\tilde{g}}^i_\delta ({\tilde{x}})={\left\{ \begin{array}{ll} {\tilde{g}}^i({\tilde{x}})&{} \quad \,\text {if } {\text {dist}}(\pi (\tilde{x}),\partial M,{\tilde{g}}^i)\ge \delta _0, \\ (\tilde{\Phi }_i)_*(\gamma ({\tilde{g}}^i)^\delta _t+dt^2) ({\tilde{x}})&{} \quad \, \text {else}. \end{array}\right. } \end{aligned}$$

The following lemma is obtained by direct computation using Lemma 20; cp. [31, pp. 1168–1170]. For the statement, we choose a smooth reference metric $\check{g}$ on ${\tilde{M}}$ that agrees with ${\tilde{g}}$ outside of a compact set.

### Lemma 23

There holds

$$\begin{aligned} \limsup _{\delta \searrow 0}\,\sup _{i\ge 1} \big ( \delta ^{-1}\,|{\tilde{g}}^i_\delta -{\tilde{g}}^i|_{\check{g}}+| D(\check{g}){\tilde{g}}^i_\delta |_{\check{g}}+\delta \,| D^2(\check{g}){\tilde{g}}^i_\delta |_{\check{g}}\big )<\infty . \end{aligned}$$

In the next lemma, the assumption that $H(\partial M,g^i) \ge 0$ is used.

### Lemma 24

([31, Proposition 3.1]). There holds, as $\delta \searrow 0$ and uniformly for all *i*, $R({\tilde{g}}_\delta ^i)\ge -O(1)$.

### Lemma 25

Let $i\ge 1$. There holds $H(\tilde{\Sigma },{\tilde{g}}_\delta ^i)>0$ on $\tilde{\Sigma }{\setminus }\pi ^{-1}(\partial \Sigma )$ provided that $\delta >0$ is sufficiently small.

### Proof

Without loss of generality, we may assume that $\Sigma$ is a connected free-boundary hypersurface. It follows that ${\tilde{\Sigma }}\subset {\tilde{M}}$ is a connected, compact hypersurface without boundary of class $C^{1,1}$ that is smooth away from $\pi ^{-1}(\partial \Sigma )$.

Let $x_0\in \partial \Sigma$. Given $0<\delta <\delta _0$, let $U_\delta =\{{\tilde{x}}\in {\tilde{M}}:{\text {dist}}(\pi ({\tilde{x}}), x_0, g^i)<\delta \}$. We choose normal coordinates for $(\partial M,g^i|_{\partial M})$ centered at $x_0$ with induced frame $\partial _1,\,\dots ,\partial _{n-1}$ such that $\partial _{n-1}=\nu (\Sigma ,g^i)$ at $x_0$. The following error estimates are independent of the choice of $x_0$. Note that

$$\begin{aligned} {\tilde{g}}^{i}=\sum _{\ell =1}^{n-1}d_\ell ^2+dt^2+O(\delta )\qquad \text {and}\qquad \nu ({\tilde{\Sigma }},{\tilde{g}}^i)=\partial _{n-1}+O(\delta ) \end{aligned}$$

in $U_\delta$. Using Lemma 23, we obtain

$$\begin{aligned} {\tilde{g}}^{i}_\delta =\sum _{\ell =1}^{n-1}d_\ell ^2+dt^2+O(\delta )\qquad \text {and}\qquad \nu ({\tilde{\Sigma }},{\tilde{g}}^i_\delta )=\partial _{n-1}+O(\delta ). \end{aligned}$$

Using Lemma 23 again, we conclude that, on $U_\delta \cap {\tilde{\Sigma }} {\setminus } \pi ^{-1}(\partial \Sigma )$,

$$\begin{aligned} \begin{aligned} H({\tilde{\Sigma }},{\tilde{g}}^i_\delta )&= {} H({\tilde{\Sigma }}, {\tilde{g}}^{i})+\Gamma ({\tilde{g}}^i_\delta )^{n-1}_{tt}+\sum _{\ell =1}^{n-2}\Gamma ({\tilde{g}}^{i}_\delta )^{n-1}_{\ell \ell }-\Gamma ( {\tilde{g}}^{i})^{n-1}_{tt}\\ {}&\qquad -\sum _{\ell =1}^{n-2}\Gamma ({\tilde{ g}}^{i})^{n-1}_{\ell \ell }+O(\delta ). \end{aligned}\end{aligned}$$

Here, $\Gamma$ denotes a Christoffel symbol. Using Lemma 23 once more, we have

$$\begin{aligned} 2\,\Gamma ({\tilde{g}}^i_\delta )^{n-1}_{\ell \ell }=\,&2\, (\partial _\ell \,{\tilde{g}}^i_{\delta })(\partial _{n-1},\partial _\ell )-(\partial _{n-1}\,{\tilde{g}}^i_{\delta })(\partial _\ell ,\partial _\ell )+O(\delta ), \\ 2\,\Gamma ({\tilde{g}}^i)^{n-1}_{\ell \ell }=\,&2\,(\partial _\ell \,{\tilde{g}}^i)(\partial _{n-1},\partial _\ell )-(\partial _{n-1}\,{\tilde{g}}^i)(\partial _\ell ,\partial _\ell )+O(\delta ), \end{aligned}$$

for all $1\le \ell \le n-2$. Using also (9) and (10), we see that

$$\begin{aligned} 2\, \Gamma ({\tilde{g}}^i_\delta )^{n-1}_{tt}=\,&2\,(\partial _t\,{\tilde{g}}^i_{\delta })(\partial _{n-1},\partial _t)-(\partial _{n-1}\,{\tilde{g}}^i_{\delta })(\partial _t,\partial _t)+O(\delta )=O(\delta ), \\ 2\,\Gamma ({\tilde{g}}^i)^{n-1}_{tt}=\,&2\,(\partial _t\,{\tilde{g}}^i)(\partial _{n-1},\partial _t)-(\partial _{n-1}\,{\tilde{g}}^i)(\partial _t,\partial _t)+O(\delta )=O(\delta ) . \end{aligned}$$

Moreover, using (9) and (10), the same argument that led to Lemma 23 shows that

$$\begin{aligned} \begin{aligned}(\partial _\ell \,{\tilde{g}}^i_{\delta })(\partial _{n-1},\partial _\ell )&=(\partial _\ell \,{\tilde{g}}^i)(\partial _{n-1},\partial _\ell )+O(\delta )\quad \text { and}\\ (\partial _{n-1}\,{\tilde{g}}^i_{\delta })(\partial _\ell ,\partial _\ell )&=(\partial _{n-1}\,{\tilde{g}}^i)(\partial _\ell ,\partial _\ell )+O(\delta ) \end{aligned} \end{aligned}$$

for all $$1\le \ell \le n-2$$.

Since $H({\tilde{\Sigma }},{\tilde{g}}^i_\delta )=H({\tilde{\Sigma }}, {\tilde{g}}^i)$ outside of $\{{\tilde{x}}\in {\tilde{\Sigma }}:{\text {dist}}(\pi ({\tilde{x}}), \partial \Sigma , g^i)<\delta \}$, the assertion follows. $\square$

### Lemma 26

Let $i\ge 1$. For every $\delta >0$ sufficiently small, there exists a sequence $\{{\tilde{\Sigma }}^i_{\delta ,j}\}_{j=1}^\infty$ of closed hypersurfaces ${\tilde{\Sigma }}^i_{\delta ,j}\subset M{\setminus } M(\Sigma )$ of class $C^2$ with

- $H({\tilde{\Sigma }}^i_{\delta ,j}, {\tilde{g}}_\delta ^{i})>0$ for every *j* and
- $${\tilde{\Sigma }}^\delta _{i,j}\rightarrow {\tilde{\Sigma }}$$ in $$C^1$$.

### Proof

This follows by approximation using mean curvature flow as in the proof of [21, Lemma 5.6] using also Lemma 25. $\square$

Recall the open set $W\Subset M$ from Lemma 20. We choose open sets $W_1\Subset W_2\Subset M$ such that

- $$W\Subset W_1$$ and
- $${\tilde{g}}$$ is $$C^2$$ in $$M{\setminus } W_1$$.

We then choose a function $\chi \in C^\infty (M)$ with

- $$0\le \chi \le 1$$,
- $$\chi =1$$ in $$M{\setminus } W_2$$, and
- $$\chi =0$$ in $$W_1$$.

We define the Riemannian metric ${\hat{g}}^i_\delta$ on ${\tilde{M}}$ by

$$\begin{aligned} {\hat{g}}^i_\delta =\chi \,{\tilde{g}}^i+(1-\chi )\,{\tilde{g}}^i_\delta . \end{aligned}$$

Note that ${\hat{g}}^i_\delta ({\tilde{x}})={\tilde{g}}^i({\tilde{x}})$ for all ${\tilde{x}}\in {\tilde{M}}$ with ${\text {dist}}(\tilde{x},\pi ^{-1}(\partial M),{\tilde{g}}^i)\ge \delta$.

### Lemma 27

There holds, as $\delta \searrow 0$ and uniformly in *i*,

$$\begin{aligned} |{\hat{g}}^i_{\delta }- {\tilde{g}}^i|_{{\tilde{g}}^i}=o(1). \end{aligned}$$

Moreover, outside of a compact subset of ${\tilde{M}}$, ${\hat{g}}^i_\delta ={\tilde{g}}^i={\tilde{g}}$ for all *i*.

### Proof

This follows from the construction using Lemmas 20 and 23. $\square$

### Lemma 28

There holds, as $\delta \searrow 0$ and uniformly in *i*,

$$\begin{aligned} \int _{{\tilde{M}}({\tilde{\Sigma }})}(\max \{-R({\hat{g}}^i_\delta ),0\})^\frac{n}{2}\,\textrm{d}v({\hat{g}}^i_\delta )= 2\,\int _{M(\Sigma )}(\max \{-R(g^i),0\})^\frac{n}{2}\,\textrm{d}v(g^i)+o(1). \end{aligned}$$

### Proof

On the one hand, if ${\tilde{x}}\in {\tilde{M}}{\setminus } W_2$ or ${\text {dist}}({\tilde{x}},\pi ^{-1}(\partial M),{\tilde{g}}^i)\ge \delta$, we have ${\hat{g}}^{i}_\delta ({\tilde{x}})={\tilde{g}}^i({\tilde{x}})$. Consequently,

$$\begin{aligned} R({\hat{g}}^{i}_\delta )({\tilde{x}})=R({\tilde{g}}^i)(\tilde{x})=R(g^i)(\pi ({\tilde{x}})). \end{aligned}$$

On the other hand, note that, as $\delta \searrow 0$,

$$\begin{aligned} |\{{\tilde{x}}\in W_2:{\text {dist}}({\tilde{x}},\pi ^{-1}(\partial M),{\tilde{g}}^i)< \delta \}|_{{\hat{g}}^i_{\delta }}=o(1). \end{aligned}$$

Moreover, recall that, in local coordinates,

$$\begin{aligned} \begin{aligned} R({\hat{g}}^i_\delta )&= {} \sum _{a,b,k=1}^n ({\hat{g}}^i_\delta )^{ab}\bigg (\partial _k\Gamma ^k_{ab}({\hat{g}}^i_\delta )-\partial _a\Gamma ^k_{kb}({\hat{g}}^i_\delta )\\ {}&\qquad +\sum _{\ell =1}^n\big [\Gamma ^k_{k\ell }({\hat{g}}^i_\delta )\,\Gamma ^\ell _{ab}({\hat{g}}^i_\delta )-\Gamma ^k_{a\ell }({\hat{g}}^i_\delta )\,\Gamma ^\ell _{kb}({\hat{g}}^i_\delta )\big ]\bigg ). \end{aligned} \end{aligned}$$

Using Lemmas 23, 24, and 27, we conclude that, as $\delta \searrow 0$,

$$\begin{aligned} R({\hat{g}}^i_\delta )=\chi \,R({\tilde{g}}^i)+(1-\chi )\,R({\tilde{g}}^i_\delta )+O(1)\ge -O(1) \end{aligned}$$

uniformly in $$\{{\tilde{x}}\in W_2:{\text {dist}}(\tilde{x},\pi ^{-1}(\partial M),{\tilde{g}}^i)< \delta \}$$.

The assertion follows from these estimates. $\square$

### Lemma 29

Let $i\ge 1$. For every $\delta >0$ sufficiently small, $({\tilde{M}},{\hat{g}}^i_{\delta })$ has horizon boundary ${\tilde{\Sigma }}^i_{\delta }\subset {\tilde{M}}({\tilde{\Sigma }})$ homologous to ${\tilde{\Sigma }}$.

### Proof

Let $\delta >0$ be sufficiently small such that there is an integer $j_0\ge 1$ with $H({\tilde{\Sigma }}^i_{\delta ,j}, {\tilde{g}}^\delta _{i})>0$ for every $j\ge j_0$; see Lemma 26. It follows that there is an outermost closed minimal hypersurface ${\tilde{\Sigma }}^i_\delta \subset {\tilde{M}}({\tilde{\Sigma }}^i_{\delta ,j_0})$ homologous to ${\tilde{\Sigma }}^i_{\delta ,j_0}$. Since $H({\tilde{\Sigma }}^i_{\delta ,j}, {\tilde{g}}^\delta _{i})>0$ for every $j\ge j_0$, by the maximum principle, $\tilde{\Sigma }^i_\delta$ cannot touch ${\tilde{\Sigma }}^i_{\delta ,j}$ for any $j\ge j_0$. Using Lemma 26, it follows that ${\tilde{\Sigma }}^i_{\delta }\subset {\tilde{M}}({\tilde{\Sigma }})$. $\square$

### Proposition 30

There exists a sequence $\{{\tilde{g}}_i\}_{i=1}^\infty$ of $C^2$-asymptotically flat metrics of class $C^2$ on ${\tilde{M}}$ with horizon boundary ${\tilde{\Sigma }}_i\subset M({\tilde{\Sigma }})$ such that

- $${\tilde{m}}({\tilde{g}}_i)=2\,m(g)$$,
- $$|{\tilde{\Sigma }}_i|_{{\tilde{g}}_i}=2\,|\Sigma |_g+o(1)$$ as $$i\rightarrow \infty $$,
- ${\tilde{g}}_i={\tilde{g}}$ outside of a compact set, and
- $${\tilde{g}}_i\rightarrow {\tilde{g}}$$ in $$C^0({\tilde{M}})$$ as $$i\rightarrow \infty $$.

Moreover, there holds, as $i\rightarrow \infty$,

$$\begin{aligned} \int _{{\tilde{M}}({\tilde{\Sigma }})}(\max \{-R({\tilde{g}}_i),0\})^\frac{n}{2}\,\textrm{d}v({\tilde{g}}_i)= 2\,\int _{M(\Sigma )}(\max \{-R(g),0\})^\frac{n}{2}\,\textrm{d}v( g)+o(1). \end{aligned}$$

For every $\alpha \in (0,1)$, ${\tilde{\Sigma }}_i\rightarrow {\tilde{\Sigma }}$ in $C^{1,\alpha }$ with multiplicity 1.

### Proof

By Lemma 20,

$$\begin{aligned} \int _{M(\Sigma )}(\max \{-R(g^i),0\})^\frac{n}{2}\,\textrm{d}v(g^i)=\int _{M(\Sigma )}(\max \{-R(g),0\})^\frac{n}{2}\,\textrm{d}v(g)+o(1). \end{aligned}$$

Using Lemmas 28, 23, and 27, we see that, passing to a diagonal subsequence, there is a sequence $\{\delta _i\}_{i=1}^\infty$ with $\delta _i\searrow 0$ such that the metrics ${\tilde{g}}_i={\hat{g}}^i_{\delta _i}$ satisfy ${\tilde{g}}_i={\tilde{g}}$ outside of a compact set, $|{\tilde{g}}_i- {\tilde{g}}|_{{\tilde{g}}}=o(1)$, and

$$\begin{aligned} \int _{{\tilde{M}}({\tilde{\Sigma }})}(\max \{-R({\tilde{g}}_i),0\})^{\frac{n}{2}}\,\textrm{d}v({\tilde{g}}_i)= 2\,\int _{M(\Sigma )}(\max \{-R(g),0\})^\frac{n}{2}\,\textrm{d}v(g)+o(1). \end{aligned}$$

Moreover, by Lemmas 19 and 27, ${\tilde{m}}({\tilde{g}}_i)=2\,m(g)$.

By Lemma 29, $({\tilde{M}},{\tilde{g}}_i)$ has horizon boundary ${\tilde{\Sigma }}_i={\tilde{\Sigma }}^i_{\delta _i}\subset {\tilde{M}}({\tilde{\Sigma }})$. By comparison with a large coordinate hemisphere, we see that

$$\begin{aligned} \limsup _{i\rightarrow \infty }|{\tilde{\Sigma }}_i|_{{\tilde{g}}_i}<\infty . \end{aligned}$$

Moreover, we have $\pi ^{-1}(\pi ({\tilde{\Sigma }}_i))={\tilde{\Sigma }}_i$. In fact, by area-minimization, there is a closed embedded minimal hypersurface that encloses $\pi ^{-1}(\pi ({\tilde{\Sigma }}_i))$. Since ${\tilde{\Sigma }}_i$ is outermost, this minimal surface coincides with ${\tilde{\Sigma }}_i$.

Fix $\alpha \in (0,1)$. By [21, Regularity Theorem 1.3 (ii)], Lemma 23, and compactness, it follows that, passing to another subsequence if necessary, ${\tilde{\Sigma }}_i$ converges to an embedded hypersurface $\tilde{\Sigma }_0\subset \tilde{M}({\tilde{\Sigma }})$ of class $C^{1,\alpha }$ in $C^{1,\alpha }$ possibly with multiplicity. By standard elliptic estimates, this convergence is smooth away from $\pi ^{-1}(\partial M)$ and there holds $H({\tilde{\Sigma }}_0,{\tilde{g}})=0$ on $\tilde{\Sigma }_0{\setminus } \pi ^{-1}(\partial M)$. Since $\pi ^{-1}(\pi ({\tilde{\Sigma }}_i))={\tilde{\Sigma }}_i$, there holds ${\tilde{\Sigma }}_i{\setminus } \pi ^{-1}(\partial M)={\tilde{\Sigma }}_i^+\cup {\tilde{\Sigma }}_i^-$ with $\pi ({\tilde{\Sigma }}_i^\pm )=\pi ({\tilde{\Sigma }}_i)$ and ${\tilde{\Sigma }}_i^+\cap {\tilde{\Sigma }}^-_i=\emptyset$. Let $\Sigma _i=\pi ({\tilde{\Sigma }}_i^+)$. By Lemma 29, $\Sigma _i$ is homologous to $\Sigma$ in $M(\Sigma )$ and $\partial \Sigma _i$ is homotopy equivalent to $\partial \Sigma$ in $M(\Sigma )\cap \partial M$. Since $\Sigma$ is area minimizing in $M(\Sigma )$, we have, using also Lemma 19,

11 $$\begin{aligned} \liminf _{i\rightarrow \infty }|{\tilde{\Sigma }}_i|_{{\tilde{g}}_i}\ge 2\,\liminf _{i\rightarrow \infty }|\Sigma _i|_g\ge 2\,|\Sigma |_{g}=|{\tilde{\Sigma }}|_{{\tilde{g}}}. \end{aligned}$$

Passing to a further subsequence if necessary, we have $\Sigma _i\rightarrow \Sigma _0\subset M(\Sigma )$ in $C^{1,\alpha }$ possibly with multiplicity, where $\Sigma _0$ satisfies $H(\Sigma _0,g)=0$ on $\Sigma _0{\setminus } \partial \Sigma _0$. Since $M(\Sigma )$ is an exterior region, it follows that $\Sigma _0=\Sigma$ and ${\tilde{\Sigma }}_0={\tilde{\Sigma }}$. Since ${\tilde{\Sigma }}_i$ is area-minimizing in $M({\tilde{\Sigma }}_i)$, we conclude that

$$\begin{aligned} \limsup _{i\rightarrow \infty }|{\tilde{\Sigma }}_i|_{{\tilde{g}}_i}\le |{\tilde{\Sigma }}|_{{\tilde{g}}}. \end{aligned}$$

In particular, using also (11), we see that ${\tilde{\Sigma }}_i$ converges to $\tilde{\Sigma }$ with multiplicity one.

The assertion follows. $\square$

## Conformal Transformation to Non-negative Scalar Curvature

In this section, we assume that ${\tilde{M}}$ is a smooth manifold of dimension $3\le n\le 7$ and that ${\tilde{g}}$ is a Riemannian metric on *M* of class $C^0$. We also assume that ${\tilde{g}}$ is $C^2$-asymptotically flat and that there is a closed separating hypersurface ${\tilde{\Sigma }}\subset {\tilde{M}}$ of class $C^{1,1}$. Moreover, we assume that $\{{\tilde{g}}_i\}_{i=1}^\infty$ is a sequence of Riemannian metrics ${\tilde{g}}_i$ on ${\tilde{M}}$ of class $C^2$ with the following properties.

- $({\tilde{M}},{\tilde{g}}_i)$ is $C^2$-asymptotically flat with horizon boundary ${\tilde{\Sigma }}_i\subset {\tilde{M}}({\tilde{\Sigma }})$.
- $${\tilde{m}}({\tilde{g}}_i)={\tilde{m}}({\tilde{g}})+o(1)$$ as $$i\rightarrow \infty $$.
- $$|{\tilde{\Sigma }}_i|_{{\tilde{g}}_i}=|{\tilde{\Sigma }}|_{{\tilde{g}}}+o(1)$$ as $$i\rightarrow \infty $$.
- $${\tilde{g}}_i\rightarrow {\tilde{g}}$$ in $$C^0(M)$$ as $$i\rightarrow \infty $$.
- For every $\alpha \in (0,1)$, ${\tilde{\Sigma }}_i\rightarrow {\tilde{\Sigma }}$ in $C^{1,\alpha }$ as $i\rightarrow \infty$.

Finally, we assume that, as $i\rightarrow \infty$,

12 $$\begin{aligned} \int _{{\tilde{M}}({\tilde{\Sigma }}_{i})}(\max \{0,-R({\tilde{g}}_i)\})^\frac{n}{2}\,\textrm{d}v({\tilde{g}}_i)=o(1) \end{aligned}$$

and that, for some $\tau >(n-2)/2$,

$$\begin{aligned} \sup _{i\ge 1}\,\limsup _{{\tilde{x}}\rightarrow \infty } \left[ |{\tilde{x}}|_{\bar{g}}^{\tau }\,|{\tilde{g}}_i-{\bar{g}}|_{{\bar{g}}}+|{\tilde{x}}|^{\tau +1}_{\bar{g}}\,|D({\bar{g}}){\tilde{g}}_i|_{{\bar{g}}}+|{\tilde{x}}|^{\tau +2}_{\bar{g}}\,|D^2({\bar{g}}){\tilde{g}}_i|_{{\bar{g}}}\right] <\infty . \end{aligned}$$

In this section, we construct Riemannian metrics ${\hat{g}}_i$ conformally related to ${\tilde{g}}_i$ which have non-negative scalar curvature.

### Proposition 31

For every *i* sufficiently large, there exists a function $\tilde{u}_i\in C^\infty ({\tilde{M}}({\tilde{\Sigma }}_i))$ such that the Riemannian metric

$$\begin{aligned} {\hat{g}}_i={\tilde{u}}_i^{\frac{4}{n-2}}\,{\tilde{g}}_i \end{aligned}$$

has the following properties.

- $$R({\hat{g}}_i)\ge 0$$ in $${\tilde{M}}({\tilde{\Sigma }}_i)$$.
- $$H({\tilde{\Sigma }}_i,{\hat{g}}_i)=0$$.
- $({\tilde{M}},{\hat{g}}_i)$ is $C^2$-asymptotically flat with ${\tilde{m}}({\hat{g}}_i)={\tilde{m}}({\tilde{g}})+o(1)$ as $i\rightarrow \infty$.
- $${\hat{g}}_i\rightarrow {\tilde{g}}$$ in $$C^0({\tilde{M}}({\tilde{\Sigma }}))$$ as $$i\rightarrow \infty $$.
- For every $\alpha \in (0,1),$ ${\tilde{u}}_i\rightarrow 1$ in $C_{loc}^{1,\alpha }({\tilde{M}}({\tilde{\Sigma }}))$ as $i\rightarrow \infty$.

### Proof

This has been proved in [31, §4.1] in the special case where $\tilde{\Sigma }_i={\tilde{\Sigma }}=\emptyset$. Compared to [31, (35)], we take ${\tilde{u}}_i\in C^\infty ({\tilde{M}}({\tilde{\Sigma }}))$ to be the unique solution of

13 $$\begin{aligned} {\left\{ \begin{array}{ll} -\frac{4\,(n-1)}{n-2} \Delta ({\tilde{g}}_i)\,{\tilde{u}}_i-\max \{-R({\tilde{g}}_i),0\}\,{\tilde{u}}_i=0\qquad &{}\text {in }{\tilde{M}}({\tilde{\Sigma }}_i),\qquad \qquad \qquad \qquad \\ D({\tilde{g}}_i)_{\nu ({\tilde{\Sigma }}_i,{\tilde{g}}_i)}{\tilde{u}}_i=0 &{}\text {on }{\tilde{\Sigma }}_i,\text { and}\\ \lim _{{\tilde{x}}\rightarrow \infty } {\tilde{u}}_i=1. \end{array}\right. } \end{aligned}$$

As shown in [36, Lemma 3.2], the existence of such a solution follows from (12) and the fact that, for every $\alpha \in (0,1)$, ${\tilde{\Sigma }}_i$ is of class $C^{1,\alpha }$. We may now repeat the proofs of [31, Lemma 4.1, Proposition 4.1, and Lemma 4.2]; the only difference is that the elliptic estimates for the function ${\tilde{u}}_i$ now also depend on estimates on the $C^{1,\alpha }$-regularity of ${\tilde{\Sigma }}_i$, which, by assumption, are uniform in *i*. By Lemma 44, we have that $H({\tilde{\Sigma }}_i,{\hat{g}}_i)=0$. $\square$

### Lemma 32

$({\tilde{M}},{\hat{g}}_i)$ has horizon boundary $\hat{\Sigma }_i\subset {\tilde{M}}({\tilde{\Sigma }}_i)$ and there holds $|\hat{\Sigma }_i|_{{\hat{g}}_i}\ge |{\tilde{\Sigma }}_i|_{{\tilde{g}}_i}-o(1)$ as $i\rightarrow \infty$.

### Proof

Using $H({\tilde{\Sigma }}_i,{\hat{g}}_i)=0$, it follows that $(\tilde{M},{\hat{g}}^i)$ has horizon boundary $\hat{\Sigma }_i\subset \tilde{M}({\tilde{\Sigma }}_i)$. The assertion now follows from Proposition 31, using that ${\tilde{\Sigma }}_i$ is area-minimizing in ${\tilde{M}}({\tilde{\Sigma }}_i)$ with respect to ${\tilde{g}}_i$. $\square$

### Lemma 33

Suppose that there is a map ${\tilde{F}}:{\tilde{M}}\rightarrow {\tilde{M}}$ with the following properties.

- ${\tilde{F}}$ is an isometry with respect to ${\tilde{g}}_i$ for every *i*.
- ${\tilde{S}}=\{{\tilde{x}}\in {\tilde{M}}:{\tilde{F}}({\tilde{x}})={\tilde{x}}\}$ is a separating hypersurface such that ${\tilde{M}}{\setminus } {\tilde{S}}=M^+\cup M^-$ with $M^+\cap M^-=\emptyset$.
- ${\tilde{g}}$ is $C^2$ in $M^\pm$ and $M^\pm \cap {\tilde{\Sigma }}\subset M^\pm$ is an outermost minimal hypersurface.

Then $$ |\hat{\Sigma }_i|_{{\hat{g}}_i}\le |{\tilde{\Sigma }}_i|_{{\tilde{g}}_i}+o(1). $$

### Proof

This follows as in the proof of Proposition 30. $\square$

### Remark 34

In the situation of Section 3, we may take ${\tilde{F}}:{\tilde{M}}\rightarrow {\tilde{M}}$ to be the unique map with $\pi \circ {\tilde{F}}=\pi$ and ${\tilde{F}}\ne {\text {Id}}$.

## Proof of Theorem 8

Let (*M*, *g*) be an asymptotically flat half-space of dimension $3\le n\le 7$ with horizon boundary $\Sigma \subset M$ and such that $R(g)\ge 0$ in $M(\Sigma )$ and $H(\partial M,g)\ge 0$ on $M(\Sigma )\cap \partial M$.

### Proof of Theorem 8

Using Proposition 16 to obtain a conformally flat approximation of (*M*, *g*), Proposition 30 to double the approximation, and Proposition 31, Lemmas 32, and 33 to conformally transform the double to non-negative scalar curvature, we see that there exists a smooth manifold ${\tilde{M}}$ of dimension *n* and a sequence $\{{\hat{g}}_i\}_{i=1}^\infty$ of Riemannian metrics ${\hat{g}}_i$ on ${\tilde{M}}$ with the following properties.

- $({\tilde{M}},{\hat{g}}_i)$ is asymptotically flat with horizon boundary ${\hat{\Sigma }}_{i}\subset {\tilde{M}}$
- $$R({\hat{g}}_i)\ge 0$$ in $${\tilde{M}}({\hat{\Sigma }}_i)$$
- $${\tilde{m}}({\hat{g}}_i)=2\,m(g)+o(1)$$ as $$i\rightarrow \infty $$
- $$|{\hat{\Sigma }}_{i}|_{{\hat{g}}_i}=2\,|\Sigma |_{g}+o(1)$$ as $$i\rightarrow \infty $$

The assertion follows. $\square$

## Mass-Decreasing Variations and Rigidity

Let (*M*, *g*) be an asymptotically flat half-space of dimension $3\le n\le 7$ with horizon boundary $\Sigma \subset M$ such that $R(g)\ge 0$ in $M(\Sigma )$ and $H(\partial M,g)\ge 0$ on $M(\Sigma )\cap \partial M$. We also assume that equality holds in (4), i.e. , that

$$\begin{aligned} m(g)=\left( \frac{1}{2}\right) ^{\frac{n}{n-1}}\,\left( \frac{|\Sigma |_g}{\omega _{n-1}}\right) ^{\frac{n-2}{n-1}}. \end{aligned}$$

The goal of this section is to show that $(M(\Sigma ),g)$ is isometric to the exterior region of a Schwarzschild half-space (3).

The argument in Lemma 35 below is modeled on the proof of [36, Corollary 3.1], where Schoen and Yau study the equality case of the positive mass theorem.

### Lemma 35

(Cp.  [36, Corollary 3.1]). There holds $R(g)=0$ in $M(\Sigma )$.

### Proof

Suppose, for a contradiction, that $R(g)\ne 0$. Recall the definition (34) of the weighted Hölder space $C^{2,\alpha }_{\tau }(M(\Sigma ))$. By Proposition 47, there is a unique solution $v\in C_\tau ^{2,\alpha }(M(\Sigma ))$ of

$$\begin{aligned} \begin{aligned} {\left\{ \begin{array}{ll} -\frac{4\,(n-1)}{(n-2)}\Delta _{g}\,v+R(g)\,(1+v)=0\qquad &{}{}\text{ in } {\text{ int }}(M(\Sigma )) ,\qquad \qquad \qquad \qquad \\ \ D(g)_{\nu (\partial M,g)}v=0&{}{}\text{ on } M(\Sigma )\cap \partial M, \text{ and } \\ v=0&{}{} \text{ on } \Sigma . \end{array}\right. } \end{aligned} \end{aligned}$$

Since $R(g)\ne 0$, *v* is non-constant. By the maximum principle, we have $-1<v<0$ in $M(\Sigma ){\setminus } \Sigma$ and $D(g)_{\nu (\Sigma ,g)}v<0$ on $\Sigma$. We define the family $\{g_t\}_{t\in [0,1)}$ of Riemannian metrics

$$\begin{aligned} g_t=(1+t\,v)^{\frac{4}{n-2}}\,g \end{aligned}$$

on $M(\Sigma )$. Note that $g_t$ is asymptotically flat for every $t\in [0,1)$. Moreover, by Lemma 44, we have, for every $t\in (0,1)$,

- $$R(g_t)\ge 0$$ in $$M(\Sigma )$$,
- $$H(\partial M,g_t)\ge 0$$ on $$M(\Sigma )\cap \partial M$$, and
- $$H(\Sigma ,g_t)>0$$.

Arguing as in the proof of Proposition 16, we find that $(M,g_t)$ has horizon boundary $\Sigma _t\subset M(\Sigma )$ and that $\Sigma _t\rightarrow \Sigma$ smoothly as $t\searrow 0$. Using that $v=0$ on $\Sigma$ and that $H(\Sigma ,g)=0$, we conclude that

$$\begin{aligned} \lim _{t\searrow 0}t^{-1}\,\big (|\Sigma _t|_{g_t}-|\Sigma |_g\big )=0. \end{aligned}$$

Next, we compute $m(g_t)$ in the asymptotically flat chart of (*M*, *g*). By (2),

$$\begin{aligned} \begin{aligned}&2\,(n-1)\,\omega _{n-1}\,m(g_t)\\&=\lim _{\lambda \rightarrow \infty }\lambda ^{-1}\,\bigg (\sum _{i,\,j=1}^n\int _{ {\mathbb {R}}^n_+\cap {S}^{n-1}_\lambda (0)}(1+t\,v)^{\frac{4}{n-2}}\,x^i\,\big [(\partial _jg)(e_i,e_j)-(\partial _ig)(e_j,e_j)\big ]\,\textrm{d}\mu ({\bar{g}})\\ {}&\qquad \qquad +\sum _{i=1}^{n-1}\int _{({\mathbb {R}}^{n-1}\times \{0\})\cap S^{n-1}_\lambda (0)} (1+t\,v)^{\frac{4}{n-2}}\,x^i\,g(e_i,e_n)\,\textrm{d}l({\bar{g}}) \bigg )\\ {}&\qquad \qquad +\frac{4}{n-2}\,t\,\lim _{\lambda \rightarrow \infty }\lambda ^{-1}\,\sum _{i,\,j=1}^n\int _{ {\mathbb {R}}^n_+\cap {S}^{n-1}_\lambda (0)}(1+t\,v)^{\frac{6-n}{n-2}}\,x^i\,\\&\qquad \qquad \qquad \quad \big [\partial _jv\,g(e_i,e_j)-\partial _iv\,g(e_j,e_j)\big ]\,\textrm{d}\mu ({\bar{g}}). \end{aligned}\end{aligned}$$

Using that *g* is asymptotically flat (1) and that $v\in C_{\tau }^{2,\alpha }(M(\Sigma ))$, we have

$$\begin{aligned} \lim _{\lambda \rightarrow \infty }\lambda ^{-1}\,\sum _{i,\,j=1}^n\int _{ {\mathbb {R}}^n_+\cap {S}^{n-1}_\lambda (0)}v\,x^i\,\big [(\partial _jg)(e_i,e_j)-(\partial _ig)(e_j,e_j)\big ]\,\textrm{d}\mu ({\bar{g}})=0 \end{aligned}$$

and

$$\begin{aligned} \lim _{\lambda \rightarrow \infty }\lambda ^{-1}\sum _{i=1}^{n-1}\int _{({\mathbb {R}}^{n-1}\times \{0\})\cap S^{n-1}_\lambda (0)} v\,x^i\,g(e_i,e_n)\,\textrm{d}l({\bar{g}}) =0. \end{aligned}$$

It follows that

$$\begin{aligned} \lim _{t\searrow 0} t^{-1}\,\big (m(g_t)-m(g)\big )=-\frac{2}{(n-2)\,\omega _{n-1}}\,\lim _{\lambda \rightarrow \infty }\lambda ^{-1}\, \sum _{i=1}^n\int _{ {\mathbb {R}}^n_+\cap {S}^{n-1}_\lambda (0)}x^i\,\partial _iv\,\textrm{d}\mu ({\bar{g}}). \end{aligned}$$

In conjunction with Lemma 51, we conclude that

$$\begin{aligned} \lim _{t\searrow 0} t^{-1}\,\big (m(g_t)-m(g)\big )<0 \end{aligned}$$

and, in particular, that

$$\begin{aligned} \lim _{t\searrow 0} t^{-1}\,\bigg [m(g_t)-\left( \frac{1}{2}\right) ^{\frac{n}{n-1}}\,\left( \frac{|\Sigma _t|_{g_t}}{\omega _{n-1}}\right) ^{\frac{n-2}{n-1}} -m(g)+\left( \frac{1}{2}\right) ^{\frac{n}{n-1}}\,\left( \frac{|\Sigma |_{g}}{\omega _{n-1}}\right) ^{\frac{n-2}{n-1}}\bigg ]<0. \end{aligned}$$

As this is not compatible with Corollary 10, the assertion follows. $\square$

### Lemma 36

There holds $H(\partial M,g)=0$ on $M(\Sigma )\cap \partial M$.

### Proof

Suppose, for a contradiction, that there is $U\Subset M(\Sigma ){\setminus } \Sigma$ open with $U\cap \partial M\ne \emptyset$ such that $H(\partial M,g)>0$ on $U\cap \partial M$.

By Lemma 49, there exists $\psi \in C^\infty (M)$ with the following properties.

- $\psi$ has compact support in $U\cap \partial M$.
- $\Delta _g\psi \ge 0$ in $M(\Sigma )$ and $\Delta _g\psi > 0$ at some point.

Let $0<t_0<\sup _{x\in M}|\psi |$. We define the family $\{g_t\}_{t\in [0,t_0)}$ of Riemannian metrics

$$\begin{aligned} g_t=(1-t\,\psi )^{\frac{4}{n-2}}\,g. \end{aligned}$$

By Lemmas 44 and 35, there holds $R(g_t)\ge 0$ in $M(\Sigma )$ and $R(g_t)> 0$ at some point for every $t\in (0,t_0)$. Decreasing $t_0>0$ if necessary, we also have $H(\partial M,g_t)\ge 0$ on $M(\Sigma )\cap \partial M$ for every $t\in (0,t_0)$.

On the one hand, arguing as in the proof of Proposition 16, $(M,g_t)$ has horizon boundary $\Sigma _t\subset M(\Sigma )$ with $\Sigma _t\rightarrow \Sigma$ smoothly as $t\searrow 0$. Using that $g_t=g$ near $\Sigma$ and that $M(\Sigma )$ is an exterior region, we conclude that $\Sigma _t=\Sigma$ for every $t\in (0,t_0)$ sufficiently small. On the other hand, clearly, $m(g_t)=m(g)$ for every $t\in (0,t_0)$. It follows that

$$\begin{aligned} m(g_t)=\left( \frac{1}{2}\right) ^{\frac{n}{n-1}}\,\left( \frac{|\Sigma _t|_{g_t}}{\omega _{n-1}}\right) ^{\frac{n-2}{n-1}} \end{aligned}$$

for every $t\in (0,t_0)$ sufficiently small. As this is not compatible with Lemma 35, the assertion follows. $\square$

The following lemma is the key technical step in the proof of Theorem 12.

### Lemma 37

Suppose that there is $U\Subset M(\Sigma ){\setminus } \Sigma$ open with $U\cap \partial M\ne \emptyset$ such that $h(\partial M,g)\ne 0$ on $U\cap \partial M$. There exists a smooth family $\{g_t\}_{t\in [0,t_0)}$ of Riemannian metrics $g_t$ on *M* such that

- $g_t=g$ outside of *U* and $g_0=g$,
- $$\lim _{t\searrow 0} t^{-1}(|U\cap \partial M|_{g_t}-|U\cap \partial M|_g)=0$$, and
- $$\lim _{t\searrow 0} t^{-1}\,H(\partial M, g_t)= 0$$ on $$ M(\Sigma )\cap \partial M$$.

Moreover,

$$\begin{aligned} \begin{aligned} \lim _{t\searrow 0}\,t^{-1}\,R(g_t)\ge 0 \end{aligned} \end{aligned}$$

with strict inequality at some point.

### Proof

Let $x_0\in \partial M{\setminus } \Sigma$ be such that $h(\partial M,g)(x_0) \ne 0$. Using that $H(\partial M,g)=0$ on $M(\Sigma )\cap \partial M$, see Lemma 36, we see that there is an orthonormal basis $e_1,\,e_2,\dots ,e_{n-1}$ of $T_{x_0}(\partial M)$ of principal directions of $h(\partial M,g)$ with

14 $$\begin{aligned}{} & {} h(\partial M,g)(x_0)(e_1,e_1)\!\!\ge \!\! \max \{|h(\partial M,g)(x_0)(e_2,e_2)|,\dots ,|h(\partial M,g)(x_0)(e_{n-1},e_{n-1})|\},\nonumber \\{} & {} h(\partial M,g)(x_0)(e_1,e_1)>0,\quad \text{ and }\quad -h(\partial M,g)(x_0)(e_2,e_2)> 0. \end{aligned}$$

Given $\varepsilon >0$ sufficiently small, we define a local parametrization

$$\begin{aligned} \Psi _\varepsilon :\{y\in {\mathbb {R}}^{n-1}:|y|_{\bar{g}}<\varepsilon \}\rightarrow \partial M \qquad \text {given by} \qquad \Psi _\varepsilon (y)=\exp (g|_{\partial M})_{x_0}\bigg (\sum _{\ell =1}^{n-1}y^\ell \,e_\ell \bigg ) \end{aligned}$$

of $\partial M$ near $x_0$. Decreasing $\varepsilon >0$ if necessary, we obtain a local parametrization

$$\begin{aligned} \Phi _\varepsilon :\{y\in {\mathbb {R}}^{n-1}:|y|_{\bar{g}}<\varepsilon \}\times \{s\in {\mathbb {R}}:0\le s< \varepsilon \}\rightarrow M \end{aligned}$$

of *M* near $x_0$ given by

$$\begin{aligned} \Phi _\varepsilon (y,s)=\exp (g)_{\Psi _\varepsilon (y)}(s\,\nu (\partial M,g)(\Psi _\varepsilon (y))). \end{aligned}$$

By construction,

15 $$\begin{aligned} \Phi _\varepsilon ^*g=\gamma _s+ds^2 \end{aligned}$$

where, for each $s\in [0,\varepsilon )$, $\gamma _s$ is a Riemmanian metric on $\{y\in {\mathbb {R}}^{n-1}:|y|_{{\bar{g}}}<\varepsilon \}$. Note that for all $s\in [0,\varepsilon ]$, as $\varepsilon \searrow 0$,

16 $$\begin{aligned} \begin{aligned}&\bullet \qquad \gamma _s={\bar{g}}|_{{\mathbb {R}}^{n-1}}+o(1),\qquad \qquad \qquad \qquad \qquad \\ {}&\bullet \qquad D({\bar{g}})\gamma _s=O(1), \text{ and }\\ {}&\bullet \qquad D^2({\bar{g}})\gamma _s=O(1). \end{aligned} \end{aligned}$$

Moreover, computing the Christoffel symbols of *g* in the chart $\Phi _{\varepsilon }$, we see that, as $\varepsilon \searrow 0$,

17 $$\begin{aligned} \sup _{s\in [0,\varepsilon )}\,\sup _{\,|y|_{\bar{g}}<\varepsilon }\bigg |\frac{1}{2}\, (D(\bar{g})_{e_n}\gamma _s)(y)+h(\partial M,g)(\Psi _\varepsilon (y))\bigg |_{{\bar{g}}}=o(1). \end{aligned}$$

In particular,

18 $$\begin{aligned} \sup _{s\in [0,\varepsilon )}\,\sup _{\,|y|_{\bar{g}}<\varepsilon }{\text {tr}}_{{\bar{g}}|_{{\mathbb {R}}^{n-1}}}(D(\bar{g})_{e_n}\gamma _s)(y)=o(1). \end{aligned}$$

By (14), there holds on $\Phi ( {\mathbb {R}}^n_+\cap B^n_\varepsilon (0))$ for all $1\le i,\, j\le n-1$ with $i\ne j$, as $\varepsilon \searrow 0$,

19 $$\begin{aligned} \begin{aligned} h(\partial M,g)(e_1,e_1)\ge \,&|h(\partial M,g)(e_i,e_i)|+o(1), \\ |h(\partial M,g)(e_i,e_j)|=\,&o(1),\\ h(\partial M,g)(e_1,e_1)\ge \,&h(\partial M,g)(x_0)(e_1,e_1)-o(1), \text { and} \\ -h(\partial M,g)(e_2,e_2)\ge \,&0. \end{aligned} \end{aligned}$$

Let $K>1$ large be a constant to be chosen later, $\rho \in C^\infty ({\mathbb {R}}^{n-1})$ be the function from Lemma 50, and $\delta \in (0,\varepsilon )$. We define $\rho _\varepsilon \in C^\infty ({\mathbb {R}}^{n-1})$ by $\rho _\varepsilon (y)=\rho (\varepsilon ^{-1}\,y)$ and

20 $$\begin{aligned} \eta _{\delta }:[0,\infty )\rightarrow {\mathbb {R}}\qquad \text { by}\qquad \eta _\delta (s)={\left\{ \begin{array}{ll} \exp ^{-(1-\delta ^{-1}\,s)^{-1}}\qquad \,\,\,\,&{}\text {if}\qquad 0\le s<\delta ,\\ 0&{}\text {else}. \end{array}\right. } \end{aligned}$$

Let

$$\begin{aligned} f_{\varepsilon ,\delta }(y,s)=\eta _\delta (s)\,\rho _\varepsilon (y). \end{aligned}$$

By the Gram-Schmidt process, given $(y,s)\in {\mathbb {R}}^{n-1}\times {\mathbb {R}}$ with $|y|_{\bar{g}}<\varepsilon$ and $0\le s<\varepsilon$, there exists an invertible $(n-1)\times (n-1)$-matrix $A=A(y,s)$ smoothly depending on (*y*, *s*) such that $\gamma _s=A^t\,A$. Moreover, as $\varepsilon \searrow 0$,

21 $$\begin{aligned} A={\text {Id}}+o(1),\qquad D({\bar{g}})A=O(1),\qquad \text {and}\qquad D^2({\bar{g}})A=O(1). \end{aligned}$$

Let $E:{\mathbb {R}}^{n-1}\rightarrow {\mathbb {R}}^{n-1}$ be the linear map given by $E(e_1)=-e_1$, $E(e_2)=e_2$, and $E(e_i)=0$ when $3\le i\le n-1$. We define a symmetric (0, 2)-tensor $\sigma ^{\varepsilon ,\delta }$ on ${\mathbb {R}}^n_+\cap B^n_\varepsilon (0)$ by

$$\begin{aligned} \begin{aligned}&\sigma ^{\varepsilon ,\delta }|_{{\mathbb {R}}^{n-1}}=f_{\varepsilon ,\delta }\,A^t\,E\,A,\\&\sigma ^{\varepsilon ,\delta }(e_n,e_i)=\sigma ^{\varepsilon ,\delta }(e_i,e_n)=0 \text { for }\,i=1,\,2,\dots ,n-1, \text { and} \\&\sigma ^{\varepsilon ,\delta }(e_n,e_n)=-K\,f_{\varepsilon ,\delta }. \end{aligned} \end{aligned}$$

Note that

22 $$\begin{aligned} {\text {tr}}_{\Phi _\varepsilon ^*g}\sigma ^{\varepsilon ,\delta }=-K\,f_{\varepsilon ,\delta } \end{aligned}$$

and, for each $s\in [0,\varepsilon )$,

23 $$\begin{aligned} {\text {tr}}_{\gamma _s}(\sigma ^{\varepsilon ,\delta }|_{{\mathbb {R}}^{n-1}})=0. \end{aligned}$$

For $t_0>0$ sufficiently small, we obtain a family $\{g^{\varepsilon ,\delta }_t\}_{t\in [0,t_0)}$ of Riemannian metrics $g^{\varepsilon ,\delta }_t$ on *M* where

$$\begin{aligned} g^{\varepsilon ,\delta }_t(x)={\left\{ \begin{array}{ll}g(x)+t\,((\Phi _\varepsilon )_*\sigma ^{\varepsilon ,\delta })(x)&{} \qquad \text {if } x\in {\text {Im}}(\Phi _{\varepsilon }),\\ g(x)&{}\qquad \text {else}. \end{array}\right. } \end{aligned}$$

Note that

$$\begin{aligned} \Phi ^*_\varepsilon g_t^{\varepsilon ,\delta }=\gamma _s+ds^2+t\,\sigma ^{\varepsilon ,\delta }. \end{aligned}$$

Moreover, by (23),

$$\begin{aligned} \lim _{t\searrow 0} t^{-1}(|U\cap \partial M|_{g^{\varepsilon ,\delta }_t}-|U\cap \partial M|_g)=0. \end{aligned}$$

We estimate the linearization of $R(g^{\varepsilon ,\delta }_t)$ at $t=0$. All geometric expressions below are computed in the chart $\Phi _\varepsilon$. Recall from [22, (6.7)] that

24 $$\begin{aligned} \lim _{t\searrow 0}\,t^{-1}\,R(g^{\varepsilon ,\delta }_t)={\text {div}}_g{\text {div}}_g\sigma ^{\varepsilon ,\delta }-\Delta _g{\text {tr}}_g\sigma ^{\varepsilon ,\delta }-g({\text {Ric}}(g),\sigma ^{\varepsilon ,\delta }). \end{aligned}$$

By (22), (15), and (18), we have, as $\varepsilon \searrow 0$,

$$\begin{aligned} \begin{aligned}\Delta _g{\text{ tr }}_g\sigma ^{\varepsilon ,\delta }&=-K\,\eta ''_\delta \,\rho _\varepsilon +K\,\eta _\delta '\,\rho _{\varepsilon }\,\sum _{i,\,j=1}^{n-1}g^{ij}\,\Gamma (g)^n_{ij} -K\,\eta _\delta \,\sum _{i,j=1}^{n-1}g^{ij}\,\left[ \partial _i\partial _j\rho _{\varepsilon }-\sum _{\ell =1}^{n-1}\Gamma (g)^\ell _{ij}\,\partial _\ell \rho _{\varepsilon }\right] \\ {}&=\,-K\,\eta ''_\delta \,\rho _\varepsilon -K\,\eta _\delta \,\sum _{i,j=1}^{n-1}\left[ g^{ij}\,\partial _i\partial _j\rho _{\varepsilon }-\sum _{\ell =1}^{n-1}g^{ij}\,\Gamma (g)^\ell _{ij}\,\partial _\ell \rho _{\varepsilon }\right] +K\,o(\eta _\delta '\,\rho _{\varepsilon }).\end{aligned} \end{aligned}$$

Moreover,

$$\begin{aligned} g({\text {Ric}}(g),\sigma ^{\varepsilon ,\delta })=K\,O(f_{\varepsilon ,\delta }). \end{aligned}$$

Next, we compute

$$\begin{aligned}&{\text{ div }}_g\,{\text{ div }}_g\,{\sigma ^{\varepsilon ,\delta }}\\\quad&=\sum _{a,\,b,\,i,\,j=1}^n\bigg [g^{ab}\,g^{ij}\,\partial _i\partial _a \sigma ^{\varepsilon ,\delta }_{jb}+g^{ab}\,\partial _a g^{ij}\,\partial _i \sigma ^{\varepsilon ,\delta }_{jb}\\ {}&\qquad -\sum _{\ell =1}^n\bigg (g^{ab}\,g^{ij}\,\Gamma (g)^\ell _{ij}\,\partial _a\sigma ^{\varepsilon ,\delta }_{\ell b} +g^{ab}\,g^{ij}\,\Gamma (g)^{\ell }_{ib}\,\partial _a\sigma ^{\varepsilon ,\delta }_{j\ell }+g^{ab}\,g^{ij}\,\Gamma (g)^{\ell }_{ab}\,\partial _i\sigma ^{\varepsilon ,\delta }_{j\ell }\bigg )\bigg ]\\ {}&\qquad +K\,O(f_{\varepsilon ,\delta }) \end{aligned}$$

Using (15), (16), and (21), we have

$$\begin{aligned}{} & {} \sum _{a,\,b,\,i,\,j=1}^n g^{ab}\,g^{ij}\,\partial _i\partial _a \sigma ^{\varepsilon ,\delta }_{jb}\\{} & {} \quad =-K\,\eta ''_\delta \,\rho _\varepsilon +O(\eta _\delta \,D^2(\bar{g}|_{{\mathbb {R}}^{n-1}})\rho _{\varepsilon })+O(\eta _\delta \,D(\bar{g}|_{{\mathbb {R}}^{n-1}})\rho _{\varepsilon })+O(f_{\varepsilon ,\delta }) \end{aligned}$$

and, using also (18),

$$\begin{aligned}&\sum _{a,\,b,\,i,\,j=1}^n\bigg [ g^{ab}\,\partial _a g^{ij}\,\partial _i \sigma ^{\varepsilon ,\delta }_{jb}-\sum _{\ell =1}^n\bigg ( g^{ab}\,g^{ij}\,\Gamma (g)^\ell _{ij}\,\partial _a\sigma ^{\varepsilon ,\delta }_{\ell b}+g^{ab}\,g^{ij}\,\Gamma (g)^{\ell }_{ab}\,\partial _i\sigma ^{\varepsilon ,\delta }_{j\ell }\bigg )\bigg ]\\ {}&\qquad =2\,K\,\eta '_\delta \,\rho _{\varepsilon }\,\sum _{i,\,j=1}^{n-1}g^{ij}\,\Gamma (g)^n_{ij} +O(\eta _\delta \,D(\bar{g}|_{{\mathbb {R}}^{n-1}})\rho _{\varepsilon })+O(f_{\varepsilon ,\delta }) \\&\qquad =K\,o(\eta '_\delta \,\rho _{\varepsilon })+O(\eta _\delta \,D({\bar{g}}|_{{\mathbb {R}}^{n-1}})\rho _{\varepsilon })+O(f_{\varepsilon ,\delta }). \end{aligned}$$

Likewise, using also (17) and (19),

$$\begin{aligned}&g^{ab}\,g^{ij}\,\Gamma (g)^{\ell }_{ib}\,\partial _a\sigma ^{\varepsilon ,\delta }_{j\ell }=\,[h(\partial M,g)(e_1,e_1)-h(\partial M,g)(e_2,e_2)]\,\eta '_\delta \,\rho _\varepsilon \\&\qquad +o(\eta '_\delta \,\rho _{\varepsilon })+O(\eta _\delta \,D(\bar{g}|_{{\mathbb {R}}^{n-1}})\rho _{\varepsilon })+O(f_{\varepsilon ,\delta }). \end{aligned}$$

We conclude that, as $\varepsilon \searrow 0$,

$$\begin{aligned} \begin{aligned}&\lim _{t\searrow 0}\,t^{-1}\,R(g^{\varepsilon ,\delta }_t)\\ {}&\qquad \ge -[h(\partial M,g)(e_1,e_1)-h(\partial M,g)(e_2,e_2)]\,\eta '_\delta \,\rho _\varepsilon \\ {}&\qquad \qquad +K\,\eta _\delta \,\sum _{i,j=1}^{n-1}\left[ g^{ij}\,\partial _i\partial _j\rho _{\varepsilon }-\sum _{\ell =1}^{n-1}g^{ij}\,\Gamma (g)^\ell _{ij}\,\partial _\ell \rho _{\varepsilon }\right] \\ {}&\qquad \qquad -K\,o(\eta '_\delta \,\rho _{\varepsilon })-O(\eta _\delta \,D^2(\bar{g}|_{{\mathbb {R}}^{n-1}})\rho _{\varepsilon })-O(\eta _\delta \,D(\bar{g}|_{{\mathbb {R}}^{n-1}})\rho _{\varepsilon })-K\,O(f_{\varepsilon ,\delta }). \end{aligned} \end{aligned}$$

By Lemma 50 and (16), we may choose $K>1$ such that, for all $y\in {\mathbb {R}}^{n-1}$ with $\varepsilon /2\le |y|_{{\bar{g}}}<\varepsilon$ and $s\in [0,\delta )$,

$$\begin{aligned} \begin{aligned}&K\,\eta _\delta \,\sum _{i,j=1}^{n-1}\left[ g^{ij}\,\partial _i\partial _j\rho _{\varepsilon }-\sum _{\ell =1}^{n-1}g^{ij}\,\Gamma (g)^\ell _{ij}\,\partial _\ell \rho _{\varepsilon }\right] \\ {}&\qquad \ge O(\eta _\delta \,D^2(\bar{g}|_{{\mathbb {R}}^{n-1}})\rho _{\varepsilon }) +\,O(\eta _\delta \,D(\bar{g}|_{{\mathbb {R}}^{n-1}})\rho _{\varepsilon })+K\,O(f_{\varepsilon ,\delta }). \end{aligned}\end{aligned}$$

provided that $\varepsilon >0$ is sufficiently small. Moreover, by (19), we have, for all $y\in {\mathbb {R}}^{n-1}$ with $|y|_{{\bar{g}}}<\varepsilon$ and $s\in [0,\delta )$,

25 $$\begin{aligned} - [h(\partial M,g)(e_1,e_1)-h(\partial M,g)(e_2,e_2)]\,\eta '_\delta \,\rho _\varepsilon -K\, o(\eta '_\delta \,\rho _{\varepsilon })>0 \end{aligned}$$

provided that $\varepsilon >0$ is sufficiently small. Consequently, for all $y\in {\mathbb {R}}^{n-1}$ with $\varepsilon /2\le |y|_{\bar{g}}<\varepsilon$ and $s\in [0,\delta )$,

$$\begin{aligned} \lim _{t\searrow 0}\,t^{-1}\,R(g^{\varepsilon ,\delta }_t)\ge 0 \end{aligned}$$

provided that $\varepsilon >0$ is sufficiently small. Finally, by (20) we have $\eta _\delta =o(\eta '_\delta )$ as $\delta \searrow 0$ and, by Lemma 50, we have

$$\begin{aligned} \liminf _{\varepsilon \searrow 0}\sup \{\rho _{\varepsilon }(y):y\in {\mathbb {R}}^{n-1}\text { and }|y|_{{\bar{g}}}\le \varepsilon /2\}>0. \end{aligned}$$

Using (25), we conclude that, for all $y\in {\mathbb {R}}^{n-1}$ with $|y|_{\bar{g}}\le \varepsilon /2$ and $s\in [0,\delta )$,

$$\begin{aligned} \lim _{t\searrow 0}\,t^{-1}\,R(g^{\varepsilon ,\delta }_t)> 0 \end{aligned}$$

provided that $\varepsilon >0$ and $\delta \in (0,\varepsilon )$ are sufficiently small.

Next, we compute the linearization of $H(\partial M, g^{\varepsilon ,\delta }_t)$ at $t=0$. As before, all geometric expressions are computed in the chart $\Phi _\varepsilon$. The argument that led to (17) also shows that

$$\begin{aligned} h(\partial M,g^{\varepsilon ,\delta }_t)(\Psi _\varepsilon )=-\frac{1}{2}\, \big (D({\bar{g}})_{e_n}\gamma _s+t\,(D({\bar{g}})_{e_n} \sigma ^{\varepsilon ,\delta })|_{{\mathbb {R}}^{n-1}}\big )\big |_{s=0} \end{aligned}$$

and

$$\begin{aligned} H(\partial M,g^{\varepsilon ,\delta }_t)=-\frac{1}{2}\,{\text {tr}}_{\bar{g}|_{{\mathbb {R}}^{n-1}}}\left[ (\gamma _0+t\,\sigma ^{\varepsilon ,\delta }|_{{\mathbb {R}}^{n-1}})^{-1}\,\big (D(\bar{g})_{e_n}\gamma _s+t\,(D(\bar{g})_{e_n}\sigma ^{\varepsilon ,\delta })|_{{\mathbb {R}}^{n-1}}\big )\big |_{s=0}\right] . \end{aligned}$$

Using that $\gamma _s=A^t\,A$ and that $\sigma ^{\varepsilon ,\delta }|_{{\mathbb {R}}^{n-1}}=A^T\,E\,A$, we obtain

$$\begin{aligned} \begin{aligned}&H(\partial M,g_t^{\varepsilon ,\delta })\\ {}&\quad =-\frac{1}{2}\,{\text{ tr }}_{\bar{g}|_{{\mathbb {R}}^{n-1}}}\left[ (A^t\,({\text{ Id }}+t\,f_{\varepsilon ,\delta }\,E)\,A)^{-1}\,D(\bar{g})_{e_n}(A^t\,({\text{ Id }}+t\,f_{\varepsilon ,\delta }\,E)\,A)\right] \big |_{s=0} \\ {}&\quad = -\frac{1}{2}\,{\text{ tr }}_{\bar{g}|_{{\mathbb {R}}^{n-1}}}\left[ (A^t)^{-1}\,D(\bar{g})_{e_n}A^t+A^{-1}\,D(\bar{g})_{e_n}A\right. \\ {}&\quad \qquad \left. +\,({\text{ Id }}+t\,f_{\varepsilon ,\delta }\,E)^{-1}\,t\,(D(\bar{g})_{e_n} f_{\varepsilon ,\delta })\,E\right] \big |_{s=0}. \end{aligned} \end{aligned}$$

Note that

$$\begin{aligned} \frac{d}{dt}\bigg |_{t=0}{\text {tr}}_{\bar{g}|_{{\mathbb {R}}^{n-1}}}\left[ ({\text {Id}}+t\,f_{\varepsilon ,\delta }\,E)^{-1}\,t\,\,E\right] ={\text {tr}}_{\bar{g}|_{{\mathbb {R}}^{n-1}}}[E]=0. \end{aligned}$$

We conclude that

$$\begin{aligned} \frac{d}{dt}\bigg |_{t=0} H(\partial M,g^{\varepsilon ,\delta }_t)=0. \end{aligned}$$

The assertion follows. $\square$

### Lemma 38

There holds $h(\partial M,g)=0$ on $M(\Sigma )\cap \partial M$.

### Proof

Suppose, for a contradiction, that there is $U\Subset M(\Sigma ){\setminus } \Sigma$ open with $U\cap \partial M\ne \emptyset$ such that $h(\partial M,g)\ne 0$ on $U\cap \partial M$. Let $\{g_t\}_{t\in [0,t_0)}$ be the family of Riemannian metrics on *M* from Lemma 37. Arguing as in the proof of [2, Lemma 4.3], using Proposition 47 instead of [2, Proposition 3.3], we find that, for all $t\ge 0$ sufficiently small, there is a unique solution $u_t\in C^{2,\alpha }(M)$ of

26 $$\begin{aligned} \begin{aligned} {\left\{ \begin{array}{ll} -\frac{4\,(n-1)}{(n-2)}\Delta _{g_t}\,u_t+R(g_t)\,u_t=0\qquad &{}{}\text{ in } {\text{ int }}(M(\Sigma )),\\ \frac{2\,(n-1)}{n-2}\,D(g)_{\nu (\partial M,g_t)}u_t+H(\partial M,g_t)\,u_t=0&{}{}\text{ on } M(\Sigma )\cap \partial M, \\ u_t=1&{}{} \text{ on } \Sigma , \end{array}\right. } \end{aligned}\end{aligned}$$

such that $(u_t-1)\in C_{\tau }^{2,\alpha }(M(\Sigma ))$. Moreover, the limit

$$\begin{aligned} {\dot{u}}=\lim _{t\searrow 0}\,t^{-1}\,(u_t-1) \end{aligned}$$

exists in $C_{\tau }^{2,\beta }(M(\Sigma )$ for every $\beta \in (0,\alpha )$; see [36, pp. 73-74]. By (26),

$$\begin{aligned} \begin{aligned} {\left\{ \begin{array}{ll} -\frac{4\,(n-1)}{(n-2)}\Delta _{g}\,{\dot{u}}+\lim _{t\searrow 0} \,t^{-1}\,R(g_t)=0\qquad &{}{}\text{ in } {\text{ int }}(M(\Sigma )),\qquad \qquad \qquad \qquad \\ D(g)_{\nu (\partial M,g)}{\dot{u}}^i=0&{}{}\text{ on } M(\Sigma )\cap \partial M, \\ \ {\dot{u}}=0&{}{} \text{ on } \Sigma . \end{array}\right. } \end{aligned}\end{aligned}$$

Let

$$\begin{aligned} {\hat{g}}_t=(1+u_t)^\frac{4}{n-2}\,g_t. \end{aligned}$$

Using that $(u_t-1)\in C_\tau ^{2,\alpha }(M(\Sigma ))$, we see that ${\hat{g}}_t$ is $C^2$ −asymptotically flat and, using also Lemma 44, that $R({\hat{g}}_t)= 0$ in $M(\Sigma )$ and $H(\partial M,{\hat{g}}_t)= 0$ on $M(\Sigma )\cap \partial M$. Using Lemma 37, we see that ${\dot{u}}$ is non-constant. By the maximum principle, ${\dot{u}}<0$ in $M(\Sigma ){\setminus } \Sigma$ and $D(g)_{\nu (\Sigma ,g)}{\dot{u}}<0$ on $\Sigma$. Now, Lemmas 44 and 37 imply that $H(\Sigma ,{\hat{g}}_t)>0$ for all $t>0$ sufficiently small. As in the proof of Proposition 16, it follows that $(M,{\hat{g}}_t)$ has horizon boundary $\hat{\Sigma }_t\subset M(\Sigma )$ and that $\hat{\Sigma }_t\rightarrow \Sigma$ smoothly as $t\searrow 0$.

On the one hand, using that $g_t=g$ on $\Sigma$, ${\dot{u}}=0$ on $\Sigma$, and $H(\Sigma ,g)=0$, we conclude that

27 $$\begin{aligned} \lim _{t\searrow 0} t^{-1}\,\big (|{\hat{\Sigma }}_t|_{{\hat{g}}_t}-|\Sigma |_g\big )=0. \end{aligned}$$

Moreover, arguing as in the proof of Lemma 35, we have

$$\begin{aligned} \lim _{t\searrow 0}t^{-1}\,(m({\hat{g}}_t)-m(g))=-\frac{2}{(n-2)\,\omega _{n-1}}\,\lim _{\lambda \rightarrow \infty }\lambda ^{-1}\,\sum _{i=1}^n\int _{ {\mathbb {R}}^n_+\cap {S}^{n-1}_\lambda (0)}x^i\,\partial _i\dot{u}\,\textrm{d}\mu ({\bar{g}}). \end{aligned}$$

In conjunction with Lemma 51, we conclude that

28 $$\begin{aligned} \lim _{t\searrow 0}t^{-1}\,(m({\hat{g}}_t)-m(g))<0. \end{aligned}$$

On the other hand, by Corollary 10, we have

$$\begin{aligned} \lim _{t\searrow 0}\,t^{-1}\,\bigg (m({\hat{g}}_t)-\left( \frac{1}{2}\right) ^{\frac{n}{n-1}}\,\left( \frac{|{\hat{\Sigma }}_t|_{{\hat{g}}_t}}{\omega _{n-1}}\right) ^{\frac{n-2}{n-1}}-m(g)+\left( \frac{1}{2}\right) ^{\frac{n}{n-1}}\,\left( \frac{| \Sigma |_{ g}}{\omega _{n-1}}\right) ^{\frac{n-2}{n-1}}\bigg )\ge 0 \end{aligned}$$

This is not compatible with (27) and (28).

The assertion follows. $\square$

### Proof of Theorem 12

Suppose that (*M*, *g*) is an asymptotically flat half-space with horizon boundary $\Sigma \subset M$ with $R(g)\ge 0$ in $M(\Sigma )$ and $H(\partial M,g)\ge 0$ on $M(\Sigma )\cap \partial M$ such that

$$\begin{aligned} m(g)=\left( \frac{1}{2}\right) ^{\frac{n}{n-1}}\,\left( \frac{|\Sigma |_{g}}{\omega _{n-1}}\right) ^{\frac{n-2}{n-1}}. \end{aligned}$$

Recall the definitions (7) of the doubled manifold $({\tilde{M}},{\tilde{g}})$ and (8) of the projection $\pi :{\tilde{M}}\rightarrow M$. Moreover, recall that ${\tilde{\Sigma }}=\pi ^{-1}(\Sigma )$. By Lemma 38, $h(\partial M,g)=0$ on $M(\Sigma )\cap \partial M$. In particular, $({\tilde{M}}({\tilde{\Sigma }}),{\tilde{g}})$ is a $C^2$-asymptotically flat manifold with mass ${\tilde{m}}({\tilde{g}})=2\,m(g)$ and ${\tilde{\Sigma }}$ is a closed minimal surface with $|{\tilde{\Sigma }}|_{{\tilde{g}}}=2\,|\Sigma |_{g}$. By symmetry, using that $M(\Sigma )$ is an exterior region, it follows that ${\tilde{M}}({\tilde{\Sigma }})$ is an exterior region. By Theorem 39, $({\tilde{M}}({\tilde{\Sigma }}),{\tilde{g}})$ is isometric to the exterior region of the Schwarzschild space (31) with mass $\tilde{m}({\tilde{g}})$. It follows that $(M(\Sigma ),g)$ is isometric to the exterior region of the Schwarzschild half-space (3) of mass *m*(*g*). $\square$

## Rigidity in the Riemannian Penrose Inequality

In this section, we give an argument alternative to that in [26] to show that the assumption that $({\tilde{M}},{\tilde{g}})$ be spin in the rigidity statement of [8, Theorem 1.4], stated here as Theorem 41, is not necessary.

For the statement of Theorem 39 below, recall from Appendix A the definition of an asymptotically flat manifold $({\tilde{M}},{\tilde{g}})$, of its horizon boundary ${\tilde{\Sigma }}$, and of the exterior region $M({\tilde{\Sigma }})$.

### Theorem 39

Let $({\tilde{M}},{\tilde{g}})$ be an asymptotically flat manifold of dimension $3\le n\le 7$ with horizon boundary ${\tilde{\Sigma }}\subset {\tilde{M}}$ such that $R({\tilde{g}})\ge 0$ in $M({\tilde{\Sigma }})$ and

$$\begin{aligned} {\tilde{m}}({\tilde{g}})=\frac{1}{2}\,\left( \frac{|{\tilde{\Sigma }}|_{{\tilde{g}}}}{\omega _{n-1}}\right) ^{\frac{n-2}{n-1}}. \end{aligned}$$

Then $({\tilde{M}}({\tilde{\Sigma }}),{\tilde{g}})$ is isometric to the exterior region of a Schwarzschild space (31).

### Proof

Following the argument given in [8, §6], we aim to show that the manifold $({\hat{M}},{\hat{g}})$ obtained by reflection of $({\tilde{M}},{\tilde{g}})$ across ${\tilde{\Sigma }}$ is smooth so that the characterization of equality in the positive mass theorem, stated here as Theorem 40, applies to $({\hat{M}},{\hat{u}}^{\frac{4}{n-2}}\,{\hat{g}})$. Here, ${\hat{u}}\in C^2({\hat{M}})$ is the unique harmonic function that approaches 1 respectively 0 in the two ends of $({\hat{M}},{\hat{g}})$. To this end, it suffices to show that ${\tilde{\Sigma }}$ is totally geodesic.

The argument presented in Lemma 35 shows that $R({\tilde{g}})=0$. If $h({\tilde{\Sigma }},{\tilde{g}})\ne 0$, the argument presented in Lemma 37 shows that there exists a family $\{{\tilde{g}}_t\}_{t\in [0,t_0)}$ of Riemannian metrics on $({\tilde{M}},{\tilde{g}})$ such that

- ${\tilde{g}}_t={\tilde{g}}$ outside of a compact set,
- $${\tilde{g}}_t\rightarrow {\tilde{g}}$$ smoothly as $$t\searrow 0$$,
- $$\lim _{t\searrow 0} t^{-1}(|{\tilde{\Sigma }}|_{{\tilde{g}}_t}-|{\tilde{\Sigma }}|_{{\tilde{g}}})=0$$,
- $$\lim _{t\searrow 0} t^{-1}\,H({\tilde{\Sigma }}, {\tilde{g}}_t)= 0$$,

and

$$\begin{aligned} \begin{aligned} \lim _{t\searrow 0}\,t^{-1}\,R({\tilde{g}}_t)\ge 0 \text { with strict inequality at some point}. \end{aligned} \end{aligned}$$

Adapting the argument in the proof of Lemma 38 to the case of an asymptotically flat manifold, we see that this leads to a contradiction with the inequality in Theorem 41. $\square$

## Figures and Tables

**Fig. 4:**
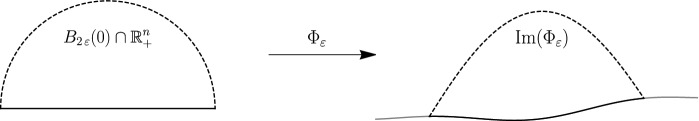
An illustration of the map $\Phi _\varepsilon$. $B^n_{2\,\varepsilon }(0)\cap \partial {\mathbb {R}}^n_+$ is presented by the solid black line on the left. The map $\Phi _\varepsilon$ maps $B^n_{2\,\varepsilon }(0)\cap \partial {\mathbb {R}}^n_+$ to a subset of $U\cap \partial M$ which is presented by the solid black line on the right. $\partial M$ is showed by the solid gray line

## A. Asymptotically Flat Manifolds

In this section, we recall some facts about asymptotically flat manifolds.

Let $3\le n\le 7$. A metric ${\tilde{g}}$ on $\{{\tilde{x}}\in {\mathbb {R}}^n:|{\tilde{x}}|_{{\bar{g}}}>1/2\}$ is called $C^2$-asymptotically flat if its scalar curvature is integrable and if there is $\tau >(n-2)/2$ such that, as ${\tilde{x}}\rightarrow \infty$,

29 $$\begin{aligned} \begin{aligned}&|{\tilde{g}}-{\bar{g}}|_{{\bar{g}}}+|x|_{{\bar{g}}}\,|D({\bar{g}}){\tilde{g}}|_{\bar{g}}+|x|^2_{{\bar{g}}}\,|D^2({\bar{g}}){\tilde{g}}|_{{\bar{g}}}=O\left( |\tilde{x}|_{{\bar{g}}}^{-\tau }\right) . \end{aligned} \end{aligned}$$

A complete connected Riemannian manifold $({\tilde{M}},{\tilde{g}})$ of dimension *n* is said to be an asymptotically flat manifold if the following properties all hold.

- ${\tilde{g}}$ is of class $C^2$.
- There is a non-empty compact subset of ${\tilde{M}}$ whose complement is diffeomorphic to the set $\{{\tilde{x}}\in {\mathbb {R}}^n:|{\tilde{x}}|_{{\bar{g}}}>1/2\}$.
- The pull-back of ${\tilde{g}}$ by this diffeomorphism is $C^2$-asymptotically flat.

We usually fix such a diffeomorphism and refer to it as the asymptotically flat chart. The mass of an asymptotically flat manifold is the quantity

30 $$\begin{aligned} {\tilde{m}}({\tilde{g}})=\lim _{\lambda \rightarrow \infty } \frac{1}{2\,(n-1)\,\omega _{n-1}}\,\lambda ^{-1}\,\sum _{i,\,j=1}^n&\int _{S^{n-1}_\lambda (0)}\tilde{x}^i\,[(\partial _j\tilde{g})\big (e_i,e_j)-(\partial _i\tilde{g})(e_j,e_j)\big ]\,\textrm{d}\mu (\bar{g}); \end{aligned}$$

see [4, p. 999]. Here, $e_1,\dots ,e_n$ are the canonical basis vectors of ${\mathbb {R}}^n$ and $\omega _{n-1}=|S^{n-1}_1(0)|_{{\bar{g}}}$ denotes the area of the $(n-1)$-dimensional unit sphere. Bartnik has showed that the mass (30) of a $C^2$-asymptotically flat manifold converges and does not depend on the choice of asymptotically flat chart; see [6, Theorem 4.2].

Let ${\tilde{\Sigma }}\subset M$ be a compact hypersurface without boundary. We call the components of such a hypersurface closed. If ${\tilde{\Sigma }}$ is separating, we orient ${\tilde{\Sigma }}$ by the unit normal $\nu ({\tilde{\Sigma }},{\tilde{g}})$ pointing towards the closure ${\tilde{M}}({\tilde{\Sigma }})$ of the non-compact component of $\tilde{M}{\setminus } {\tilde{\Sigma }}$. The mean curvature $H({\tilde{\Sigma }},{\tilde{g}})$ is then computed as the divergence of $-\nu ({\tilde{\Sigma }},{\tilde{g}})$ along ${\tilde{\Sigma }}$.

We say that $({\tilde{M}},{\tilde{g}})$ has horizon boundary if there is a non-empty hypersurface ${\tilde{\Sigma }}\subset {\tilde{M}}$ with the following two properties.

- Each component of ${\tilde{\Sigma }}$ is a closed minimal hypersurface.
- Every closed minimal hypersurfaces in ${\tilde{M}}({\tilde{\Sigma }})$ is a component of ${\tilde{\Sigma }}$.

If $({\tilde{M}},{\tilde{g}})$ has horizon boundary ${\tilde{\Sigma }}\subset M$, we say that ${\tilde{M}}({\tilde{\Sigma }})$ is the exterior region of ${\tilde{M}}$ and we call the horizon ${\tilde{\Sigma }}$ an outermost minimal surface. An example of an exterior region with horizon boundary is the Schwarzschild space of mass ${\tilde{m}}>0$ and dimension $n\ge 3$ defined by

31 $$\begin{aligned} ({\tilde{M}}({\tilde{\Sigma }}),{\tilde{g}})=\bigg (\bigg \{\tilde{x}\in {\mathbb {R}}^n:|{\tilde{x}}|_{{\bar{g}}}\ge \left( \frac{\tilde{m}}{2}\right) ^{\frac{1}{n-2}}\bigg \},\bigg (1+\frac{\tilde{m}}{2}\,|{\tilde{x}}|_{{\bar{g}}}^{2-n}\bigg )^\frac{4}{n-2}\,{\bar{g}}\bigg ). \end{aligned}$$

where

$$\begin{aligned} {\tilde{\Sigma }}=\left\{ {\tilde{x}}\in {\mathbb {R}}^n:|{\tilde{x}}|_{\bar{g}}=\left( \frac{{\tilde{m}}}{2}\right) ^{\frac{1}{n-2}}\right\} . \end{aligned}$$

The positive mass theorem has been proved by Schoen and Yau in [36] using minimal surface techniques and subsequently by Witten in [40] using certain solutions of the Dirac equation.

### Theorem 40

([35, Theorem 4.2]). Let $({\tilde{M}},{\tilde{g}})$ be an asymptotically flat manifold of dimension $3\le n\le 7$ whose scalar curvature is non-negative. There holds ${\tilde{m}}({\tilde{g}})\ge 0$. Moreover, ${\tilde{m}}({\tilde{g}})=0$ if and only if $({\tilde{M}},{\tilde{g}})$ is isometric to $({\mathbb {R}}^n,{\bar{g}})$.

The Riemannian Penrose inequality has been proved by Huisken and Ilmanen in the case where the horizon boundary is connected using inverse mean curvature flow in [21]. For general horizon boundary, it has been obtained by Bray [7] using his quasi-static flow. Bray’s technique has been extended to higher dimensions in his joint work [8] with Lee.

### Theorem 41

([8, Theorem 1.4]). Let $({\tilde{M}},{\tilde{g}})$ be an asymptotically flat manifold of dimension $3\le n\le 7$ with horizon boundary ${\tilde{\Sigma }}\subset {\tilde{M}}$ such that $R({\tilde{g}})\ge 0$ in $M({\tilde{\Sigma }})$. There holds

32 $$\begin{aligned} {\tilde{m}}({\tilde{g}})\ge \frac{1}{2}\,\left( \frac{|{\tilde{\Sigma }}|_{{\tilde{g}}}}{\omega _{n-1}}\right) ^{\frac{n-2}{n-1}}. \end{aligned}$$

If $({\tilde{M}},{\tilde{g}})$ is a spin manifold, equality holds if and only if $({\tilde{M}}({\tilde{\Sigma }}),{\tilde{g}})$ is isometric to the exterior region of a Schwarzschild space (31).

### Remark 42

The assumption that $({\tilde{M}},{\tilde{g}})$ be spin is not necessary; see [26, Theorem 1.1] and Theorem 39.

### Remark 43

Lam [24, Corollary 20] and Huang and Wu [20, Theorem 2] have showed that Theorem 41 holds in all dimensions if $({\tilde{M}},{\tilde{g}})$ is an asymptotically flat hypersurface of a Euclidean space.

## B. Riemannian Geometry

In this section, we recall some facts from Riemannian geometry.

### Lemma 44

([22, §3]). Let (*M*, *g*) be a Riemannian manifold of dimension $n\ge 3$ and $u\in C^\infty (M)$ be a positive function. Let

$$\begin{aligned} g^u=u^{\frac{4}{n-2}}\,g \end{aligned}$$

and suppose that $\Sigma \subset M$ is a two-sided hypersurface with unit normal $\nu (\Sigma ,g)$ and mean curvature $H(\Sigma ,g)$ computed as the divergence of $\nu (\Sigma ,g)$ along $\Sigma$.

There holds

$$\begin{aligned} R(g^u)=u^{-\frac{n+2}{n-2}}\,\left( -\frac{4\,(n-1)}{n-2}\Delta _g u+R(g)\,u\right) \end{aligned}$$

and

$$\begin{aligned} H(\Sigma ,g^u)=u^{-\frac{n}{n-2}}\left( \frac{2\,(n-1)}{n-2}\,D(g)_{\nu (\Sigma ,g)}u+H(\Sigma ,g)\,u\right) . \end{aligned}$$

### Lemma 45

Let $n\ge 2$. There exists $\varepsilon >0$ with the following property. Suppose that $\sigma$ is a symmetric (0, 2)-tensor on ${\mathbb {R}}^n_+\cap B^n_1(0)$ with

33 $$\begin{aligned} |\sigma |_{{\bar{g}}}+|D({\bar{g}})\sigma |_{{\bar{g}}}+|D^2(\bar{g})\sigma |_{{\bar{g}}}<\varepsilon \end{aligned}$$

and let $g=\bar{g}+\sigma$. The map $\{y\in \mathbb {R}^{n-1}:|y|_{\bar{g}}<1/2\}\times [0,1/2)\rightarrow \mathbb {R}^n_+\cap B^n_1(0)$ given by

$$\begin{aligned}{} & {} (y,t)\mapsto \exp (g)_{y}({t\,\nu (\partial {\mathbb {R}}^n_+,g)(y)}) \end{aligned}$$

is injective.

### Proof

Let $y_1,\,y_2\in {\mathbb {R}}^{n-1}$ with $|y_1|_{\bar{g}},\,|y_2|_{{\bar{g}}}<1/2$. Let

$$\begin{aligned} \omega _1(t)=\exp (g)_{y_1}({t\,\nu (\partial {\mathbb {R}}^n_+,g)(y_1)}) \qquad \text {and} \qquad \omega _2(t)=\exp (g)_{y_2}({t\,\nu (\partial {\mathbb {R}}^n_+,g)(y_2)}). \end{aligned}$$

Moreover, let $s\in [0,1/2]$ be maximal such that, for all $t\in [0,s]$,

$$\begin{aligned} \begin{aligned}&\bullet \qquad \omega _1(t),\,\omega _2(t)\in {\mathbb {R}}^n_+\cap B^n_{3/4}(0),\\ {}&\bullet \qquad \frac{1}{2}\,|y_1-y_2|_{{\bar{g}}}\le |\omega _1(t)-\omega _2(t)|_{{\bar{g}}}\le 2\,|y_1-y_2|_{{\bar{g}}}, \text{ and }\\ {}&\bullet \qquad |\dot{\omega }_1(t)-\dot{\omega }_2(t)|_{{\bar{g}}}\le \frac{1}{2}\,|y_1-y_2|_{{\bar{g}}}. \end{aligned} \end{aligned}$$

By (33), as $$\varepsilon \searrow 0$$,

$$\begin{aligned} |\dot{\omega }_1(0)-\dot{\omega }_2(0)|_{\bar{g}}=O(\varepsilon )\,|y_1-y_2|_{{\bar{g}}}. \end{aligned}$$

In particular, $s>0$. By the geodesic equation,

$$\begin{aligned} |\ddot{\omega }_1(t)-\ddot{\omega }_2(t)|_{\bar{g}}=O(\varepsilon )\,|y_1-y_2|_{{\bar{g}}} \end{aligned}$$

on [0, *s*]. Integrating, it follows that $s= 1/2$ provided that $\varepsilon >0$ is sufficiently small.

The assertion follows. $\square$

## C. Laplace Operator on Asymptotically Flat Half-spaces with Horizon Boundary

In [2, Proposition 3.3], Almaraz, Barbosa, and de Lima have proved an existence and uniqueness result for the Laplace equation on asymptotically flat half-spaces. In this section, we explain how their result can be adapted to an asymptotically flat half-space with horizon boundary.

Let (*M*, *g*) be an asymptotically flat half-space of rate $\tau >(n-2)/2$ with horizon boundary.

We fix an asymptotically flat chart $\Phi :\{x\in {\mathbb {R}}^n_+:|x|_{{\bar{g}}}>1/2\}\rightarrow M$. We may assume that ${\text {Im}}(\Phi )\cap \Sigma =\emptyset$. Let $K\subset M$ be the connected compact set with $\partial K=\Sigma \cup \Phi (\{x\in {\mathbb {R}}^n_+:|x|_{{\bar{g}}}\ge 2\})$.

Given $\alpha \in (0,1)$, an integer $k\ge 0$, and $u\in C_{loc}^{k,\alpha }(M)$, we adapt from [2, §3] the definition of the weighted Hölder norm

34 $$\begin{aligned} \begin{aligned}&|u|_{C^{k,\alpha }_\tau (M(\Sigma ))} \\ {}&\quad =\,|u|_{C^{k,\alpha }(K)}+\sum _{i=0}^k\sup _{|x|_{{\bar{g}}}>1}|x|_{{\bar{g}}}^{i+\tau }\,|(D^i({\bar{g}})u)(x)|_{{\bar{g}}}\\ {}&\quad \qquad +\sup _{|x_1|_{{\bar{g}}}>1}\,\,\sup _{2\,|x_2-x_1|_{{\bar{g}}}<|x_1|_{{\bar{g}}}}|x_1|_{{\bar{g}}}^{k+\tau +\alpha }\,|x_1-x_2|_{{\bar{g}}}^{-\alpha }\, |(D^k({\bar{g}})u)(x_1)-(D^k({\bar{g}})u)(x_2)|_{{\bar{g}}} \end{aligned}\nonumber \\ \end{aligned}$$

where $x,\,x_1,\,x_2\in {\mathbb {R}}^n_+$. We define

$$\begin{aligned} C^{k,\alpha }_\tau (M(\Sigma ))=\{u\in C_{loc}^{k,\alpha }(M):|u|_{C^{k,\alpha }_\tau (M(\Sigma ))}<\infty \}. \end{aligned}$$

Likewise, given $\beta \in (0,1)$, an integer $\ell \ge 1$, and $a\in C_{loc}^{\ell ,\beta }(\partial M)$, we define

$$\begin{aligned} \begin{aligned}&|a|_{C^{\ell ,\beta }_\tau ( M(\Sigma )\cap \partial M)} \\&\quad =\,|a|_{C^{\ell ,\beta }( K\cap \partial M)}+\sum _{j=0}^\ell \sup _{|y|_{{\bar{g}}}>1}|y|_{\bar{g}}^{j+\tau }\,|(D^j({\bar{g}})a)(y)|_{{\bar{g}}} \\&\qquad +\sup _{|y_1|_{\bar{g}}>1}\,\,\sup _{2\,|y_1-y_2|_{{\bar{g}}}<|y_1|_{{\bar{g}}}}|y_1|_{\bar{g}}^{\ell +\tau +\beta }\,|y_1 -y_2|_{{\bar{g}}}^{-\beta }\, |(D({\bar{g}})^\ell a)(y_1) -(D({\bar{g}})^\ell a)(y_2)|_{{\bar{g}}} \end{aligned} \end{aligned}$$

where $y,\,y_1,\,y_2\in {\mathbb {R}}^{n-1}\times \{0\}$. We define

$$\begin{aligned} \begin{aligned} C_\tau ^{\ell ,\beta }( M(\Sigma )\cap \partial M)=&{} \big \{a\in C_{loc}^{\ell ,\beta }(\partial M):|a|_{C^{\ell ,\beta }_\tau ( M(\Sigma )\cap \partial M)}<\infty \\&\quad \text{ and } D(g|_{\partial M})_{\nu (\Sigma ,g)}a=0 \text{ on } \Sigma \cap \partial M\big \}.\end{aligned} \end{aligned}$$

### Proposition 46

Let $\alpha \in (0,1)$. There exists a constant $c>0$ with the following property. Given $\psi \in C^{0,\alpha }_{\tau }(M(\Sigma ))$ and $a\in C^{1,\alpha }_{\tau }( M(\Sigma )\cap \partial M)$, there exists a unique solution $v\in C_\tau ^{2,\alpha }(M(\Sigma ))$ of

35 $$\begin{aligned} {\left\{ \begin{array}{ll} -\Delta _gv-\psi =0\qquad &{}\text {in }{\text {int}}(M(\Sigma )),\qquad \qquad \qquad \qquad \qquad \qquad \qquad \qquad \\ D(g)_{\nu (\partial M,g)}v-a=0&{}\text {on } M(\Sigma )\cap \partial M,\text { and}\\ D(g)_{\nu (\Sigma ,g)}v=0&{}\text {on } \Sigma . \end{array}\right. } \end{aligned}$$

There holds

36 $$\begin{aligned} |v|_{C_{\tau }^{2,\alpha }(M(\Sigma ))}\le c\,\big (|\psi |_{C^{0,\alpha }_{\tau }(M(\Sigma ))}+|a|_{C^{1,\alpha }_{\tau }( M(\Sigma )\cap \partial M)}\big ). \end{aligned}$$

### Proof

The case where $\Sigma =\emptyset$ has been proved in [2, Proposition 3.3].

If $\Sigma \subset M$ is a compact hypersurface whose components are closed hypersurfaces or free boundary hypersurfaces, we consider the differentiable manifold ${\hat{M}}=M(\Sigma )\times \{-1,\,1\}/\sim$ where

- $(x_1,\pm 1)\sim (x_2,\pm 1)$ if and only if $x_1=x_2$ and
- $(x_1,\pm 1)\sim (x_2,\mp 1)$ if and only if $x_1,\,x_2\in \Sigma$ and $x_1=x_2.$

We define the Riemannian metric ${\hat{g}}$ on ${\hat{M}}$ by ${\hat{g}}({\hat{x}})=g(\pi ({\hat{x}}))$ where $\pi ([(x,\pm 1)])=x$. Note that $({\hat{M}},{\hat{g}})$ has two asymptotically flat ends and that ${\hat{g}}$ is of class $C^2$ away from $\pi ^{-1}(\Sigma )$. Moreover, note that, although ${\hat{g}}$ is only Lipschitz, the coefficients of $\Delta _{{\hat{g}}}$ are still Lipschitz since $\Sigma$ is minimal.

Let $\hat{\psi }:{\hat{M}}\rightarrow {\mathbb {R}}$ be given by $\hat{\psi }({\hat{x}})=\psi (\pi ({\hat{x}}))$ and ${\hat{a}}: \partial \hat{M}\rightarrow {\mathbb {R}}$ be given by ${\hat{a}}({\hat{x}})=a(\pi ({\hat{x}}))$. Clearly, $\hat{\psi }\in C^{0,\alpha }_\tau ({\hat{M}})$. Moreover, using that $D(g|_{\partial M})_{\nu (\Sigma ,g)}a=0$ on $\Sigma \cap \partial M$, we conclude that ${\hat{a}} \in C^{1,\alpha }_{\tau }(\partial {\hat{M}})$. By [2, Proposition 3.3], there exists a unique ${\hat{v}}\in C_\tau ^{2,\alpha }({\hat{M}})$ with

37 $$\begin{aligned} {\left\{ \begin{array}{ll} -\Delta _{{\hat{g}}}{\hat{v}}-\hat{\psi }=0\qquad &{}\text {in }{\hat{M}},\qquad \qquad \qquad \qquad \qquad \qquad \qquad \qquad \qquad \\ D({\hat{g}})_{\nu (\partial {\hat{M}},{\hat{g}})}{\hat{v}}-{\hat{a}}=0&{}\text {on } \partial {\hat{M}} \end{array}\right. } \end{aligned}$$

and

$$\begin{aligned} |{\hat{v}}|_{C_{\tau }^{2,\alpha }({\hat{M}})}\le c\,\big (|\hat{\psi }|_{C^{0,\alpha }_{\tau }({\hat{M}})}+|{\hat{a}}|_{C^{1,\alpha }_{\tau }(\partial {\hat{M}})}\big ). \end{aligned}$$

Here, $c>0$ is a constant independent of $\hat{\psi }$ and ${\hat{a}}$. Moreover, by uniqueness, ${\hat{v}}([x,1])={\hat{v}}([x,-1])$ for all $x\in M$. It follows that $v:M(\Sigma )\rightarrow {\mathbb {R}}$ given by $v(x)={\hat{v}}([x,1])$ satisfies (35) and (36).

The assertion follows. $\square$

### Proposition 47

Let $\alpha \in (0,1)$ and $\chi \in C^{0,\alpha }_{\tau }(M(\Sigma ))$ with $\chi \ge 0$. There exists a constant $c>0$ with the following property. Given $\psi \in C^{0,\alpha }_{\tau }(M(\Sigma ))$ and $b\in C^{2,\alpha }( \Sigma )$ with $D(g)_{\nu (\partial M,g)}b=0$ on $\partial \Sigma$, there exists a unique solution $v\in C_\tau ^{2,\alpha }(M(\Sigma ))$ of

$$\begin{aligned} {\left\{ \begin{array}{ll} -\Delta _gv+\chi \,v-\psi =0\qquad &{}\textrm{in }\, {\text {int}}(M(\Sigma )),\qquad \qquad \\ D(g)_{\nu (\partial M,g)}v=0&{} \textrm{on }\, M(\Sigma )\cap \partial M,\textrm{and}\\ v-b=0&{} \textrm{on }\, \Sigma . \end{array}\right. } \end{aligned}$$

There holds

$$\begin{aligned} |v|_{C_{\tau }^{2,\alpha }(M(\Sigma ))}\le c\,\big (|\psi |_{C^{0,\alpha }_{\tau }(M(\Sigma ))}+|b|_{C^{2,\alpha }( \Sigma )}\big ). \end{aligned}$$

### Proof

The proof is very similar to that of Proposition 46 and we only sketch the necessary modifications. First, we consider an appropriate Dirichlet problem on the double (7) of (*M*, *g*) instead of the Neumann problem (37) on $({\hat{M}},{\hat{g}})$. Second, we use [6, Proposition 2.2] and [25, Theorem 9.2] instead of [2, Proposition 3.3]. $\square$

## D. Local Perturbations of a Riemannian Metric

In this section, we construct local perturbations of a Riemannian metric that are used in this paper.

### Lemma 48

Let (*M*, *g*) be a Riemannian manifold of dimension $n\ge 3$ with boundary $\partial M$ oriented by the unit normal $\nu (\partial M,g)$ pointing towards *M*. Let $\Sigma \subset M$ be a compact hypersurface whose components are either closed or free boundary hypersurfaces. There exists a sequence $\{\psi _i\}_{i=1}^\infty$ of functions $\psi _i\in C^\infty (M)$ with the following properties:

- $$D(g)_{\nu (\Sigma ,g)}\psi _i<0$$ on $$\Sigma $$.
- $$D(g)_{\nu (\partial M,g)}\psi _i\le 0$$ on $$\partial M$$.
- $$|\psi _i|+|D(g)\psi _i|_g+|D^2(g)\psi _i|_g=o(1)$$ in *M*, as $$i\rightarrow \infty $$.

Moreover, if $W\subset M$ is a neighborhood of $\Sigma$, then ${\text {spt}}(\psi _i)\subset W$ for all but finitely many *i*.

### Proof

Let $\alpha \in C^\infty ({\mathbb {R}})$ be such that

- $$\alpha (0)=0$$,
- $$\alpha '(0)=-1$$, and
- $$\alpha (t)=0$$ if $$|t|\ge 1$$.

Let $\beta \in C^\infty ({\mathbb {R}})$ be non-negative such that

- $$\beta (t)=1$$ if $$|t|\le 1$$ and
- $$\beta (t)=0$$ if $$|t|\ge 2$$.

Let $a,\,b:M\rightarrow {\mathbb {R}}$ be given by $a(x)={\text {dist}}(x,\Sigma ,g)$ and $b(x)={\text {dist}}(x,\partial M,g)$, respectively. Here, ${\text {dist}}(\,\cdot \,,\Sigma ,g)$ and ${\text {dist}}(\,\cdot \,,\partial M,g)$ are the signed distance functions that become positive in direction of the respective unit normals. Given an integer $i\ge 1$, we define $\psi _i:M\rightarrow {\mathbb {R}}$ by

$$\begin{aligned} \psi _i(x)=i^{-3}\,\big [\alpha (i\,a(x))+\beta (i\,a(x))\,\alpha (i\,b(x))\big ]. \end{aligned}$$

Note that $\psi _i$ is smooth provided that *i* is sufficiently large and that, as $i\rightarrow \infty$,

$$\begin{aligned} |\psi _i|+|D(g)\psi _i|_g+|D^2(g)\psi _i|_g=o(1). \end{aligned}$$

Moreover, if $W\subset M$ is a neighborhood of $\Sigma$, then ${\text {spt}}(\psi _i)\subset W$ for all but finitely many *i*.

On $\Sigma$, there holds

$$\begin{aligned} i^2\, D(g)_{\nu (\Sigma ,g)}\psi _i= -1+\alpha '(i\,b(x))\,(D(g)_{\nu (\Sigma ,g)}b)(x). \end{aligned}$$

Using that $\nu (\Sigma ,g)(y)\in T_y\partial M$ for every $y\in \partial \Sigma$, we obtain $D(g)_{\nu (\Sigma ,g)}b=0$ on $\partial \Sigma$. Consequently, $(D(g)_{\nu (\Sigma ,g)}b)(x)=O(b(x))$ on $\Sigma$. Using that $\alpha '(i\,b(x))=0$ if $i\,b(x)\ge 1$, we conclude that

$$\begin{aligned} i^{2}\, D(g)_{\nu (\Sigma ,g)}\psi _i\le -\frac{1}{2}, \end{aligned}$$

provided that *i* is sufficiently large.

On $\partial M$, we have

$$\begin{aligned} i^{2}\,D(g)_{\nu (\partial M,g)}\psi _i=[\alpha '(i\,a(x))\,(D(g)_{\nu (\partial M,g)}a)(x)-\beta (i\,a(x))] \end{aligned}$$

If $i\,a(x)\ge 1$, we have

$$\begin{aligned} i^{2}\, D(g)_{\nu (\partial M,g)}\psi _i=-\,\beta (i\,a(x))\le 0. \end{aligned}$$

If $i\,a(x)<1$, we have, as before, $(D(g)_{\nu (\partial M,g)}a)(x)=O(a(x))$ while $\beta (i\,a(x))=1$. Consequently,

$$\begin{aligned} i^{2}\, D(g)_{\nu (\partial M,g)}\psi _i\le -\frac{1}{2} \end{aligned}$$

provided that *i* is sufficiently large.

The assertion follows. $\square$

### Lemma 49

Let (*M*, *g*) be a Riemannian manifold of dimension $n\ge 3$ with boundary $\partial M$. Let $U\subset M$ open be such that $U\cap \partial M\ne \emptyset$. There exists a function $\psi \in C^\infty (M)$ with the following properties.

- $$\psi \ge 0$$ in *M* and $${\text {spt}}(\psi )\subset U$$.
- $\Delta _g\psi \ge 0$ in *M* and $\Delta _g\psi >0$ at some point.

### Proof

Note that there is a constant $c>0$ such that the following holds. For every $\varepsilon >0$ sufficiently small, there exists a map $\Phi _\varepsilon :B^n_{2\,\varepsilon }(0)\cap {\mathbb {R}}^n_+\rightarrow U$ such that

- $\Phi _\varepsilon$ is an embedding,
- $$\Phi _\varepsilon ^{-1}(U\cap \partial M)=\partial {\mathbb {R}}^n_+\cap B^n_{2\,\varepsilon }(0)$$,
- $$|\Phi _{\varepsilon }^*g-{\bar{g}}|_{{\bar{g}}}\le c\,\varepsilon $$, and
- $$|D({\bar{g}})\Phi _\varepsilon ^*g|_{{\bar{g}}}\le c$$;

see Fig. 4.

Let $f_\varepsilon :{\mathbb {R}}_+^n\rightarrow {\mathbb {R}}$ be given by

$$\begin{aligned} f_\varepsilon (x)={\left\{ \begin{array}{ll} e^{-(n+2)\,(2\,\varepsilon -|x+\varepsilon \,e_n|_{{\bar{g}}})^{-1}}\quad &{}\text {if }|x+\varepsilon \,e_n|_{{\bar{g}}}< 2\,\varepsilon ,\\ 0&{}\text {else}. \end{array}\right. } \end{aligned}$$

When $|x+\varepsilon \,e_n|_{{\bar{g}}}<2\,\varepsilon$, we compute

$$\begin{aligned} \Delta _{{\bar{g}}} f_\varepsilon&=(n+2)^2\,(2\,\varepsilon -|x+\varepsilon \,e_n|_{\bar{g}})^{-4}\,f_\varepsilon \\ {}&\qquad -2\,(n+2)\,(2\,\varepsilon -|x+\varepsilon \,e_n|_{\bar{g}})^{-3}\,f_{\varepsilon }\\ {}&\qquad -(n-1)\,(n+2)\,(2\,\varepsilon -|x+\varepsilon \,e_n|_{\bar{g}})^{-2}\,|x+\varepsilon \,e_n|^{-1}_{{\bar{g}}}\,f_{\varepsilon }. \end{aligned}$$

Since $|x+\varepsilon \,e_n|_{{\bar{g}}}\ge \varepsilon$ on ${\mathbb {R}}^n_+$, it follows that

$$\begin{aligned} \Delta _{{\bar{g}}} f_\varepsilon \ge (n+2)\,(2\,\varepsilon -|x+\varepsilon \,e_n|_{\bar{g}})^{-4}\,f_{\varepsilon }. \end{aligned}$$

Likewise,

$$\begin{aligned} |D({{\bar{g}}}) f_\varepsilon |_{{\bar{g}}}\le (n+2)\,\varepsilon ^2\,(2\,\varepsilon -|x+\varepsilon \,e_n|_{\bar{g}})^{-4}\,f_{\varepsilon } \end{aligned}$$

and

$$\begin{aligned} |D^2({\bar{g}}) f_\varepsilon |_{\bar{g}}\le \sqrt{n}\,(n+2)^2\,(2\,\varepsilon -|x+\varepsilon \,e_n|_{\bar{g}})^{-4}\,f_{\varepsilon }. \end{aligned}$$

Let $\psi _\varepsilon : M\rightarrow {\mathbb {R}}$ be given by

$$\begin{aligned} \qquad \psi _\varepsilon (x)={\left\{ \begin{array}{ll} f_\varepsilon (\Phi _\varepsilon ^{-1}(x))&{}\text {if }x\in {\text {Im}}(\Phi _\varepsilon ),\\ 0&{}\text {else}. \end{array}\right. } \end{aligned}$$

Note that $\psi _\varepsilon \in C^\infty (M)$ and ${\text {spt}}(\psi _\varepsilon )\subset U$. Moreover, $\Delta _g \psi _\varepsilon \ge 0$ and $\Delta _g \psi _\varepsilon > 0$ at some point provided that $\varepsilon >0$ is sufficiently small. The assertion follows. $\square$

### Lemma 50

There exist a function $\rho \in C^\infty ({\mathbb {R}}^{n-1})$ and a constant $c>0$ with the following properties.

- $\rho (x)=0$ for all $x\in {\mathbb {R}}^{n-1}$ with $|x|_{{\bar{g}}}\ge 1$.
- $\rho (x)>0$ for all $x\in {\mathbb {R}}^{n-1}$ with $|x|_{{\bar{g}}}< 1$.
- $|\rho (x)|+|(D({\bar{g}})\rho )(x)|_{{\bar{g}}}+|(D^2( {\bar{g}})\rho )(x)|_{{\bar{g}}}\le c\, (\Delta _{{\bar{g}}} \rho )(x)$ for all $x\in {\mathbb {R}}^{n-1}$ with $1/2<|x|_{{\bar{g}}}< 1$.

### Proof

Let $\eta \in C^\infty ({\mathbb {R}})$ be a function with

- $$\eta (s)=0$$ if $$s\ge 1$$,
- $$\eta (s)=e^{-(n+1)\,(1-s)^{-1}}$$ if $$s\in [1/2,1)$$,
- $$\eta (s)>0$$ if $$s\in (1/4,1/2)$$, and
- $$\eta (s)=1$$ if $$s\le 1/4$$.

By a direct computation as in the proof of Lemma 49, the function $\rho :{\mathbb {R}}^{n-1}\rightarrow {\mathbb {R}}$ given by $\rho (x)=\eta (|x|_{{\bar{g}}})$ satisfies the asserted properties. $\square$

## E. Asymptotic Growth Estimate for Subharmonic Functions

In this section, we derive an asymptotic growth estimate for subharmonic functions on asymptotically flat half-spaces. The corresponding estimate for subharmonic functions on asymptotically flat manifolds has been stated by Corvino in [13, p. 164] and proved in detail by Czimek in [14, Proposition 2.6]. We note that the argument in [14, Proposition 2.6] can be adapted to the setting of an asymptotically flat half-space. Below, we give a different, self-contained proof.

Note that, in some sense, Lemma 51 is a quantitative version of the Hopf boundary point lemma as stated in, e.g. , [18, Lemma 3.4].

### Lemma 51

Let $n\ge 3$ and *g* be a $C^2$-asymptotically flat metric on ${\mathbb {R}}^n_+$. Suppose that there are a negative function $u\in C^{2,\alpha }_{\tau }({\mathbb {R}}^n_+)$ and a number $\lambda _0>1$ such that

- $$\Delta _gu\ge 0$$ in $${\mathbb {R}}^n_+{\setminus } B^n_{\lambda _0}(0),$$
- $\Delta _gu$ is integrable, and
- $$D(g)_{\nu (\partial {\mathbb {R}}^n_+,g)}u=0$$ on $$ \partial {\mathbb {R}}^n_+{\setminus } B^n_{\lambda _0}(0)$$.

Then

$$\begin{aligned} \lim _{\lambda \rightarrow \infty }\lambda ^{-1}\,\sum _{i=1}^n\int _{{\mathbb {R}}^n_+\cap S^{n-1}_\lambda (0)} x^i\,\partial _iu\,\textrm{d}\mu ({\bar{g}})>0. \end{aligned}$$

### Proof

We first assume that $\Delta _gu=0$.

Let $\eta \in C^\infty ({\mathbb {R}})$ be a non-negative function with $\eta '(0)=1$ and $\eta (s)=0$ if $s\ge 1/2$. Let $f:{\mathbb {R}}^n_+{\setminus }\{0\}\rightarrow {\mathbb {R}}$ be given by

$$\begin{aligned} f(x)=-(\log |x|_{{\bar{g}}})^{-1}\,|x|_{{\bar{g}}}^{-(n-2)}+\eta (|x|_{\bar{g}}^{-1}\,x_n)\,|x|_{{\bar{g}}}^{-(n-2)-\tau }. \end{aligned}$$

We compute

$$\begin{aligned} \Delta _{{\bar{g}}}f=-(2+(n-2)\,\log |x|_{{\bar{g}}})\,(\log |x|_{\bar{g}})^{-3}\,|x|_{{\bar{g}}}^{-n}+O(|x|_{{\bar{g}}}^{-n-\tau }). \end{aligned}$$

Likewise,

$$\begin{aligned} D({\bar{g}})f=O((\log |x|_{{\bar{g}}})^{-1}\,|x|_{\bar{g}}^{-(n-1)})\qquad \text {and}\qquad D^2({\bar{g}})f=O((\log |x|_{\bar{g}})^{-1}\,|x|_{{\bar{g}}}^{-n}). \end{aligned}$$

Moreover, on $\partial {\mathbb {R}}^n_+$, we have

$$\begin{aligned} D({\bar{g}})_{e_n}f=-|x|_{{\bar{g}}}^{-(n-1)-\tau }. \end{aligned}$$

Increasing $\lambda _0>1$ if necessary, we find that $\Delta _gf<0$ in ${\mathbb {R}}^n_+{\setminus } B^n_{\lambda _0}(0)$ and $D(g)_{\nu (\partial {\mathbb {R}}^n_+,g)}f<0$ on $\partial {\mathbb {R}}^n_+{\setminus } B^n_{\lambda _0}(0)$. Let $\delta >0$ be such that $u<\delta \,f$ on ${\mathbb {R}}^n_+\cap S^n_{\lambda _0}(0)$. By the maximum principle, for every $\lambda >\lambda _0$,

$$\begin{aligned} u< \delta \,(f-\inf \{f(x):x\in {\mathbb {R}}^n_+\cap S^n_\lambda (0)\}) \end{aligned}$$

in ${\mathbb {R}}^n_+\cap ({B}^n_\lambda (0){\setminus } B^n_{\lambda _0}(0))$. Letting $\lambda \rightarrow \infty$, we conclude that

38 $$\begin{aligned} u<\delta \, f\qquad \text {in }{\mathbb {R}}^n_+{\setminus } B^n_{\lambda _0}(0). \end{aligned}$$

Note that

$$\begin{aligned}{} & {} \lambda ^{-1}\,\sum _{i=1}^n\,\int _{{\mathbb {R}}^n_+\cap S^{n-1}_\lambda (0)} x^i\,\partial _iu\,\textrm{d}\mu (\bar{g})\\{} & {} \quad =\int _{{\mathbb {R}}^n_+\cap S^{n-1}_\lambda (0)} D(g)_{\nu ({\mathbb {R}}^n_+\cap S^{n-1}_\lambda (0),g)}u\,\textrm{d}\mu (g)+O(\lambda ^{(n-2)-2\,\tau }). \end{aligned}$$

By the divergence theorem, the limit

39 $$\begin{aligned} z=\lim _{\lambda \rightarrow \infty }\lambda ^{-1}\,\sum _{i=1}^n\,\int _{{\mathbb {R}}^n_+\cap S^{n-1}_\lambda (0)} x^i\,\partial _iu\,\textrm{d}\mu ({\bar{g}}) \end{aligned}$$

exists and

40 $$\begin{aligned} \lambda ^{-1}\,\sum _{i=1}^n\,\int _{{\mathbb {R}}^n_+\cap S^{n-1}_\lambda (0)} x^i\,\partial _iu\,\textrm{d}\mu (\bar{g})=z+O(\lambda ^{(n-2)-2\,\tau }). \end{aligned}$$

Suppose, for a contradiction, that $z\le 0$. Let $w:[\lambda _0,\infty )\rightarrow {\mathbb {R}}$ be given by

$$\begin{aligned} w(\lambda )=\lambda ^{-1}\,\int _{{\mathbb {R}}^n_+\cap S^{n-1}_\lambda (0)}u\,\textrm{d}\mu ({\bar{g}}) \end{aligned}$$

and note that, using also (40),

$$\begin{aligned} \begin{aligned}w'&=(n-2)\,\lambda ^{-1}\,w+\lambda ^{-2}\,\sum _{i=1}^n\,\int _{{\mathbb {R}}^n_+\cap S^{n-1}_\lambda (0)} x^i\,\partial _iu\, \textrm{d}\mu (\bar{g})\\ {}&\le (n-2)\, \lambda ^{-1}\,w+O(\lambda ^{(n-3)-2\,\tau }). \end{aligned} \end{aligned}$$

On the one hand, using (38), we have

$$\begin{aligned} w\le -\frac{1}{2}\,\delta \,\omega _{n-1}\,(\log (\lambda ))^{-1} \end{aligned}$$

and hence $w'\le (n-2)/2\,\lambda ^{-1}\,w$ for every $\lambda \ge \lambda _0$ provided that $\lambda _0>1$ is sufficiently large. It follows that

41 $$\begin{aligned} w(\lambda )\le \lambda _0^{-(n-2)/2}\,\lambda ^{(n-2)/2}\,w(\lambda _0) \end{aligned}$$

for all $\lambda >\lambda _0$ provided that $\lambda _0>1$ is sufficiently large. On the other hand, since $u\in C^{2,\alpha }_\tau ({\mathbb {R}}^n_+)$, we have

$$\begin{aligned} w(\lambda )\ge -O(\lambda ^{(n-2)-\tau }). \end{aligned}$$

Note that this is not compatible with (41). This completes the proof in the case where $\Delta _g u=0$ in ${\mathbb {R}}^n_+{\setminus } B^n_{\lambda _0}(0)$.

Now, suppose that $\Delta _g u\ge 0$ in ${\mathbb {R}}^n_+{\setminus } B^n_{\lambda _0}(0)$ and that $\Delta _gu$ is integrable. By Proposition 47, there is $v\in C^{2,\alpha }_{\tau }({\mathbb {R}}^n_+)$ with

$$\begin{aligned} \begin{aligned} {\left\{ \begin{array}{ll} \Delta _gv=0\qquad \qquad \qquad &{}{}\text{ in } {\mathbb {R}}^n_+{\setminus } B^n_{\lambda _0}(0),\qquad \qquad \qquad \qquad \qquad \\ D(g)_{\nu (\partial M,g)}v=0&{}{}\text{ on } \partial {\mathbb {R}}^n_+{\setminus } B^n_{\lambda _0}(0), \text{ and } \\ v=u&{}{}\text{ on } {\mathbb {R}}^n_+\cap \partial B^n_{\lambda _0}(0). \end{array}\right. } \end{aligned} \end{aligned}$$

By the maximum principle, $u\le v$ in ${\mathbb {R}}^n_+{\setminus } B^n_{\lambda _0}(0)$. In particular,

$$\begin{aligned} \lambda ^{1-n}\,\int _{{\mathbb {R}}^n_+\cap S^{n-1}_\lambda (0)}(u-v)\,\text {d}\bar{\mu }(g)\le 0 \end{aligned}$$

for every $\lambda \ge \lambda _0$. Moreover, using that $u,v\in C^{2,\alpha }_{\tau }({\mathbb {R}}^n_+),$ we have

$$\begin{aligned} \lim _{\lambda \rightarrow \infty }\lambda ^{1-n}\,\int _{{\mathbb {R}}^n_+\cap S^{n-1}_\lambda (0)}(u-v)\,\text {d}\bar{\mu }(g)= 0. \end{aligned}$$

Consequently,

$$\begin{aligned} \limsup _{\lambda \rightarrow \infty } \lambda ^{-1}\,\sum _{i=1}^n\int _{{\mathbb {R}}^n_+\cap S^{n-1}_\lambda (0)}x^i\,\partial _i(u-v)\,\textrm{d}\mu ({\bar{g}})\ge 0. \end{aligned}$$

We have already showed that

$$\begin{aligned} \lim _{\lambda \rightarrow \infty } \lambda ^{-1}\,\sum _{i=1}^n\int _{{\mathbb {R}}^n_+\cap S^{n-1}_\lambda (0)}x^i\,\partial _iv\,\textrm{d}\mu ({\bar{g}})>0. \end{aligned}$$

Moreover, since $\Delta _g u$ is integrable, the argument that led to (39) shows that

$$\begin{aligned} \lim _{\lambda \rightarrow \infty } \lambda ^{-1}\,\sum _{i=1}^n\int _{{\mathbb {R}}^n_+\cap S^{n-1}_\lambda (0)}x^i\,\partial _iu\,\textrm{d}\mu ({\bar{g}}) \end{aligned}$$

exists.

The assertion follows. $\square$

## F. Scalar Curvature Rigidity Results

In this section, we give an overview of several techniques that have been used to derive scalar curvature rigidity results in mathematical relativity.

The proofs given by Schoen and Yau in [37, Theorem 1] and, independently, by Gromov and Lawson in [19, Corollary A] of the following result are in some sense a precursor to the positive mass theorem, stated here as Theorem 40.

### Theorem 52

([19, 37]). Let $n\ge 3$ be an integer and $T^n=S^1\times \cdots \times S^1$ be the torus of dimension *n*. Let *g* be a Riemannian metric on $T^n$ with $R(g)\ge 0$. Then *g* is flat.

The proofs in [19, 37] show that any Riemannian metric *g* on $T^n$ with $R(g)\ge 0$ must actually satisfy $R(g)=0$. Studying the variation of scalar curvature (24), Bourguignon had previously observed that, unless ${\text {Ric}}(g)=0$, such a metric can be perturbed to a metric of positive scalar curvature; see [22, Lemma 5.2].

Let $({\tilde{M}},{\tilde{g}})$ be an asymptotically flat manifold with $R({\tilde{g}})\ge 0$ and $m({\tilde{g}})=0$; see Appendix A. The rigidity statement in the positive mass theorem, stated here as Theorem 40, can be viewed as a generalization of Theorem 52 to non-compact spaces. By constructing a global variation of ${\tilde{g}}$, Schoen and Yau have showed that, unless ${\text {Ric}}({\tilde{g}})=0$, ${\tilde{g}}$ can be perturbed to a metric of non-negative scalar curvature and negative mass; see [36, §3]. If $({\tilde{M}},{\tilde{g}})$ is spin, the alternative argument of Witten implies the existence of certain parallel spinors. The existence of these spinors implies that $({\tilde{M}},{\tilde{g}})$ is flat; see [40, §3]. We note that Shi and Tam have adapted this argument to settings with lower regularity; see [38, §3]. Recently, Lu and Miao [26, Proposition 2.1] have extended the rigidity statement in Theorem 40 to metrics with a corner. Their proof uses an argument of McFeron and Székelyhidi [29] based on the observation that the mass is constant along Ricci flow.

If $n=3$, the proof of the positive mass theorem in [36] suggests that there are no non-compact properly embedded area-minimizing surfaces in $\tilde{M}$ unless $({\tilde{M}},{\tilde{g}})$ is flat ${\mathbb {R}}^3$. This conjecture of Schoen has been confirmed by Chodosh and the first-named author in [11, Theorem 1.6]. In the proof, they use local perturbations of ${\tilde{g}}$ to construct a local foliation of a neighborhood of such a minimal surface by non-compact properly embedded area-minimizing surfaces obtained as limits of solutions of the Plateau problem. We remark that related rigidity results that restrict the topology of a horizon boundary are known; see [16, Corollary 1.4] and the references therein.

Let $({\tilde{M}},{\tilde{g}})$ be asymptotically flat of dimension $3\le n\le 7$ with horizon boundary ${\tilde{\Sigma }}\subset {\tilde{M}}$. If $n=3$ and ${\tilde{\Sigma }}$ is connected, Huisken and Ilmanen have proved the Riemannian Penrose inequality, stated here as Theorem 41, by evolving ${\tilde{\Sigma }}$ by inverse mean curvature flow to a large coordinate sphere in the asymptotically flat chart. By an explicit calculation, they have showed that the Hawking mass of the evolving horizon is non-decreasing and, in fact, constant if $({\tilde{M}}({\tilde{\Sigma }}),{\tilde{g}})$ is scalar flat and foliated by totally umbilic constant mean curvature spheres. To prove Theorem 41 in the general case where $3\le n\le 7$ and ${\tilde{\Sigma }}$ is possibly disconnected, Bray and Lee have used a conformal flow of the metric ${\tilde{g}}$ along which the mass is non-increasing while the area of the horizon boundary remains constant. Their proof shows that the mass is, in fact, constant along this flow if and only if a suitable conformal transformation of the double of ${\tilde{M}}({\tilde{\Sigma }})$ obtained by reflection across ${\tilde{\Sigma }}$ has zero mass. If $({\tilde{M}},{\tilde{g}})$ is spin, the rigidity results in [38, §3] apply to the possibly non-smooth double. As a consequence, $({\tilde{M}}({\tilde{\Sigma }}),{\tilde{g}})$ is isometric to the exterior region of a Schwarzschild space (31). The work of Lu and Miao [26] shows that the assumption that $(\tilde{M},{\tilde{g}})$ be spin is not necessary.

Finally, in Theorem, we give a short variational proof of rigidity in the Riemannian Penrose inequality that does not require the spin assumption. To this end, we show that if equality holds in (32), then the double of $({\tilde{M}}({\tilde{\Sigma }}),{\tilde{g}})$ obtained by reflection across ${\tilde{\Sigma }}$ is smooth. In fact, we observe that if ${\tilde{\Sigma }}$ has non-vanishing second fundamental form, we can locally perturb the metric ${\tilde{g}}$ to increase $R({\tilde{g}})$ without decreasing the area of ${\tilde{\Sigma }}$. By a global conformal transformation to zero scalar curvature, we may then decrease ${\tilde{m}}({\tilde{g}})$ without decreasing the area of ${\tilde{\Sigma }}$ by much.
